# Repaired tetralogy of Fallot: the roles of cardiovascular magnetic resonance in evaluating pathophysiology and for pulmonary valve replacement decision support

**DOI:** 10.1186/1532-429X-13-9

**Published:** 2011-01-20

**Authors:** Tal Geva

**Affiliations:** 1 Department of Cardiology, Children's Hospital Boston, Department of Pediatrics, Harvard Medical School, Boston, MA, USA

## Abstract

Surgical management of tetralogy of Fallot (TOF) results in anatomic and functional abnormalities in the majority of patients. Although right ventricular volume load due to severe pulmonary regurgitation can be tolerated for many years, there is now evidence that the compensatory mechanisms of the right ventricular myocardium ultimately fail and that if the volume load is not eliminated or reduced by pulmonary valve replacement the dysfunction might be irreversible. Cardiovascular magnetic resonance (CMR) has evolved during the last 2 decades as the reference standard imaging modality to assess the anatomic and functional sequelae in patients with repaired TOF. This article reviews the pathophysiology of chronic right ventricular volume load after TOF repair and the risks and benefits of pulmonary valve replacement. The CMR techniques used to comprehensively evaluate the patient with repaired TOF are reviewed and the role of CMR in supporting clinical decisions regarding pulmonary valve replacement is discussed.

## Introduction

Although the management of tetralogy of Fallot (TOF) has evolved considerably since Blalock and Taussig described the first systemic artery-to-pulmonary artery shunt in 1945 and Lillehei and Varco reported the first repair by an open-heart procedure in 1954 [1-3], optimal surgical repair has remained elusive. While early surgical mortality decreased from 50% in the late 1950's to less than 2% in the modern surgical era [4-7], residual anatomic and hemodynamic abnormalities are nearly universal. As a result, the number of patients with repaired TOF, many of whom with considerable cardiac and non-cardiac disease burden, is growing rapidly [8,9].

Right ventricular (RV) dilation from pulmonary regurgitation (PR), residual atrial and/or ventricular septal defect, tricuspid regurgitation, right ventricular outflow tract (RVOT) aneurysm, pulmonary artery stenosis, and tachyarrhythmias are some of the abnormalities frequently encountered in patients with repaired TOF. Although the hemodynamic burden associated with these anomalies is often tolerated well during childhood and adolescence, the incidences of arrhythmias, exercise intolerance, heart failure, and death nearly triple during the third postoperative decade and afterward [8-13]. Severe chronic PR--considered an important, treatable cause of RV dilatation and failure--has been the focus of many investigations in recent years. However, despite a growing body of literature on the management of severe PR after TOF repair, debate regarding the indications, methods, and optimal timing of pulmonary valve replacement (PVR) has persisted [14-17]. Cardiovascular magnetic resonance (CMR) has emerged as an essential diagnostic tool in this patient population because it overcomes many of the limitations of echocardiography, cardiac CT, and cardiac catheterization, while also providing unique quantitative data as well as prognostic information.

This article comprises two parts. The first part reviews the pathophysiology, natural history, and clinical challenges late after TOF repair. The second part discusses the role of CMR in clinical decision making with an emphasis on its role in supporting patient selection for PVR.

## Pathophysiology of Repaired TOF

### Pulmonary Regurgitation After TOF Repair

Relief of RVOT obstruction in TOF often involves disruption of pulmonary valve integrity, which leads to PR in the majority patients. Both experimental evidence and clinical data have shown that the severity of PR can increase over time [18-20]. The degree of PR is determined by (1) regurgitation orifice area; (2) RV compliance; (3) diastolic pressure difference between the main pulmonary artery (MPA) and the RV; (4) capacitance of the pulmonary arteries; and (5) duration of diastole. Some of the key factors influencing PR volume are captured by the Torricelli principle [21]:

$$\text{PR volume}=\text{ROA}\cdot \text{C}\cdot \text{DT}\cdot {\left({\text{P}}_{\text{2}}-{\text{P}}_{\text{1}}\right)}^{0.\text{5}}$$

Where ROA = regurgitation orifice area; C = constant (empiric number); DT = diastolic time; (P_2 _- P_1_) = mean diastolic pressure difference between the MPA and RV. Unlike aortic regurgitation, the diastolic pressure difference between the MPA and the RV is small. Therefore, the PR volume is largely determined by other factors, namely the size of the regurgitation orifice (typically large after TOF repair), the capacitance of the pulmonary arteries [22], and duration of diastole (related to heart rate). Other factors such as pulmonary vascular resistance and LV function may also influence PR.

**Figure 1:**
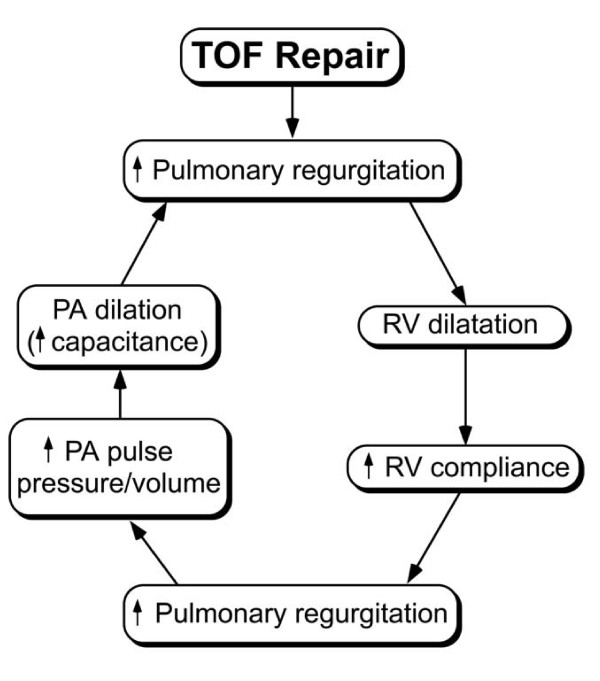
**Some of the factors influencing progression of pulmonary regurgitation after TOF repair**. This process likely plateaus at a certain point but the time course of PR has not been fully characterized.

At the time of TOF repair, the RV is hypertrophied and its compliance is low; the diameters of the central pulmonary arteries are either hypoplastic or low-normal, and their capacitance is low; and the heart rate is relatively high, which leads to a relatively short duration of diastole. Therefore, despite a relatively large regurgitation orifice immediately after TOF repair, the combination of factors described above minimizes the impact of PR. Over time, however, the increase in RV stroke volume leads to progressive rise in the size and capacitance of the central pulmonary arteries and to RV dilatation. Combined with a longer duration of diastole as heart rate decreases with age, these changes lead to progressive increase in the degree of PR (Figure 1). Experimental evidence supporting this progression was provided by Kuehne et al. who demonstrated in growing swines with a stented pulmonary valve an increase in PR fraction from 33 ± 7% at baseline to 49 ± 6% at 3 months [19]. Progression of PR, however, likely plateaus at some point but quantitative longitudinal data documenting this assumption is lacking.

### Effects of Chronic Volume Load on Ventricular Mechanics

A growing clinical experience and experimental evidence point to striking similarities between the pathophysiologic response of the left and right ventricles to severe chronic volume load. Moreover, in-vitro studies have shown similar intrinsic properties of myofibers from the left and right ventricles, including their response to pressure and volume overload conditions [23,24]. Because the pathophysiology of left ventricular (LV) response to chronic aortic regurgitation has been studied in great detail [25-27], it is worth considering the sequence of events here. Investigators divide the pathophysiologic response of the LV to severe chronic aortic regurgitation into 4 stages of variable duration: 1) A compensated stage characterized by an increase in end-diastolic volume and a combination of eccentric and concentric hypertrophy. At the cellular level, ventricular dilatation is characterized by cellular elongation, addition of myofibers, and a decrease in collagen content. At the pump level, these changes lead to an increase in end-diastolic volume and maintenance of relatively normal mass-to-volume ratio (compensated hypertrophy), end-systolic fiber stress, and global systolic function. An increase in stroke volume maintains net forward flow within normal limits. This stage may last for many years or even decades. 2) Failure of compensatory mechanisms. This stage is characterized by continued LV dilatation but with decreased mass-to-volume ratio (inadequate hypertrophy) and increased afterload (end-systolic stress). As a result, the rate and magnitude of fiber shortening decrease. This stage manifests as decreased global systolic function while intrinsic myocardial contractility remains relatively normal. 3) Reduced but reversible myocardial contractility. Over the short term, the impaired contractility may be reversible, and elimination of the volume load can result in recovery of pump function. 4) Irreversible myocardial injury associated with fibrosis and increased interstitial collagen [28]. Although valve replacement may still be tolerated and of clinical benefit [27,29], myocardial dysfunction persists.

Similar to the above-described course of LV response to volume load, patients usually tolerate chronic RV volume load without symptoms for many years. Indeed some investigators in the 1970s and 1980s considered PR benign [30]. Isolated congenital PR--a rare cardiac anomaly similar to TOF with absent pulmonary valve syndrome but without ventricular septal defect--offers a unique opportunity to examine the long-term effects of severe chronic PR without the confounding effects of cyanosis, ventricular septal defect, pulmonary stenosis, or the sequelae of cardiac surgery. In a review of 72 such cases reported in the literature [31], Shimazaki et al. found that that the annualized probability of developing symptoms of heart failure increased exponentially over time, mostly after age 40 years. Similarly, studies of patients with a secundum atrial septal defect, which also results in isolated RV volume overload, have demonstrated that the volume load adversely affects both right and left ventricular geometry and function and that these abnormalities are fully reversible when the shunt is eliminated early in life [32-36]. However, functional recovery is less complete when an atrial septal defect is closed later in life [37-39]. Taken together, these studies show that "isolated" RV volume load can be tolerated with little or no symptoms during the first 3-4 decades of life and elimination of the volume load during that period usually leads to full functional recovery. Left untreated, however, continued RV volume load is associated with an accelerated rate of progressive symptoms and incomplete functional recovery of RV systolic and diastolic function.

### RV Mechanics After TOF Repair

Although the pathophysiology of RV remodeling in response to the altered hemodynamic conditions after TOF repair is strikingly similar to the response of the LV to chronic volume load, important differences exist. Examples include chamber geometry, myofiber architecture, chamber contraction pattern, coronary artery anatomy and flow dynamic, disposition of the conduction system, and dependency on LV size and function [40]. The RV comprises two distinct embryologic components--the sinus and the infundibulum--with a complex shape. The myocardium comprises a relatively thin compact layer and a prominent layer of trabeculations interspersed with deep recesses. In contrast to the LV, the orientation of the myofibers in the RV is more horizontal and contraction is predominantly from base-to-apex (longitudinal) with a lower degree of angular motion (twist). The myocardium is supplied by a single coronary artery with nearly 50% of the flow occurring during diastole under normal conditions as oppose to ~90% in the LV. The conduction system in the RV comprises a single fascicle with a long course and a long delay in activation between the base and the distal infundibular free wall, resulting in peristalsis-like motion [41]. Although RV function impacts LV function, the reverse is much more pronounced with 63% of RV pressure rise accounted for by LV contraction.

**Figure 2:**
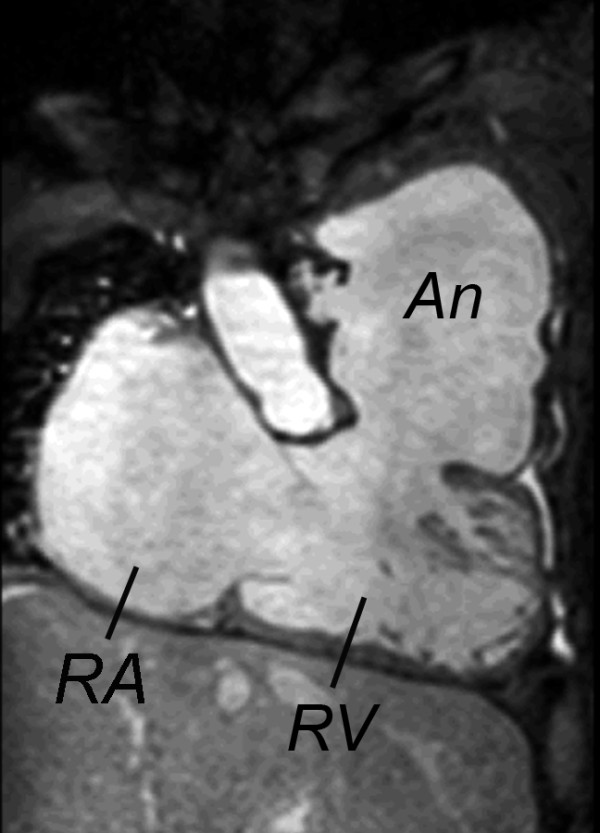
**Steady-state free precession cine CMR of the right ventricular (RV) inflow and outflow showing a large outflow tract patch aneurysm (An)**. RA = right atrium

After TOF repair, additional factors related to the operation further impact the pathophysiology. Relief of RV outflow obstruction typically involves incision of the infundibular free wall, resection of obstructive muscle bundles, disruption of the pulmonary valve with partial or complete excision, and placement of an outflow patch, which often extends across the plane of the pulmonary valve into the MPA. In some patients a conduit between the RV and the pulmonary arteries is required to provide antegrade pulmonary blood flow. The ventricular septal defect is closed with a patch, a procedure that can impair tricuspid valve function. In addition to the nearly universal occurrence of PR (discussed above), these procedures often lead to akinesis or dyskinesis of the RVOT, outflow patch aneurysm (Figure 2), fibrosis of the RV free wall, and conduction delay [42]. Table 1 summarizes the structural and functional abnormalities after TOF repair.

**Table 1:** Structural and functional abnormalities encountered in repaired TOF

Structural Abnormalities	Functional Abnormalities
Inherent to TOF repair	RV volume overload
Partial or complete removal of pulmonary valve tissue	Pulmonary regurgitation
Infundibulotomy scar	Tricuspid regurgitation
Resection of RV/infundibular muscle bundles	Left-to-right shunt
Right atriotomy scar	Ventricular septal defect
VSD patch	Atrial septal defect
Residual or recurrent lesions	Aorto-pulmonary collaterals
RV outflow tract obstruction	RV pressure overload
Main or branch pulmonary artery stenosis	RV outflow or pulmonary artery stenosis
Ventricular septal defect	Pulmonary vascular disease
Atrial septal defect	Pulmonary venous hypertension secondary to LV dysfunction
Acquired lesions	RV systolic dysfunction
Tricuspid valve abnormalities	RV diastolic dysfunction
RV outflow tract aneurysm	LV dysfunction
RV fibrosis	Ventricular conduction delay
Associated anomalies	Arrhythmias
Dilated aorta	Atrial flutter
Associated congenital cardiovascular anomalies	Atrial fibrillation
Associated genetic and non-cardiac anomalies	Ventricular tachycardia
	Co-morbidities
	Renal, pulmonary, musculoskeletal, neurodevelopmental abnormalities

Studies in animal models of RV volume load similar to conditions seen after TOF repair provide insight into myocardial adaptation. Kiriazis et al. studied an in-vitro preparation of RV papillary muscle subjected to volume load [43]. They showed that peak stress development and other markers of myocardial mechanics and energy were normal in a stage of compensated hypertrophy but reduced in uncompensated volume load. Kuehne et al. used CMR and conductance catheter techniques to investigate the effects of PR on biventricular mechanics in a growing swine model [19]. Compared with control animals, indices of RV systolic function (ejection fraction, peak dP/dt, and E_max_) were significantly reduced 3 months after induction of PR.

**Figure 3:**
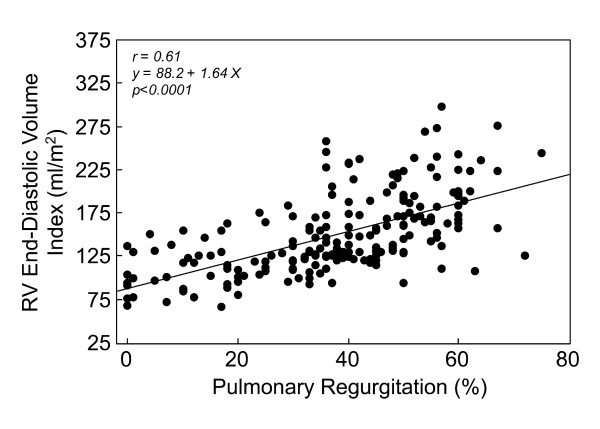
**Correlation between pulmonary regurgitation and right ventricular (RV) end-diastolic volume index in 206 patients with repaired TOF **[46].

**Figure 4:**
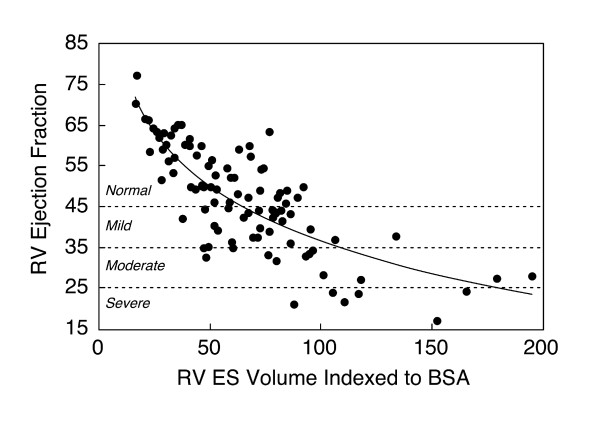
**Relationship between right ventricular end-systolic volume and RV ejection fraction in 100 patients with repaired TOF (Spearman rank correlation coefficient (r_s_) = -0.77; p < 0.001) **[10].

**Figure 5:**
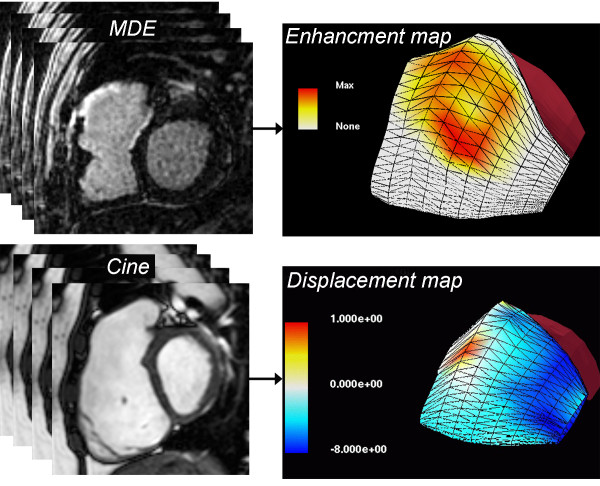
**Three-dimensional surface models of the right ventricular (RV) free wall reconstructed from multi-slice 2-dimensional short- and long-axis images**. Top panel: Scar tissue map based on late gadolinium enhancement (LGE) imaging showing extensive late hyperenhancement of the RVOT (yellow and orange). Bottom panel: Displacement map based on multi-slice cine SSFP showing dyskinesis of the RVOT (red). See Wald et al. for further technical details [42].

**Figure 6:**
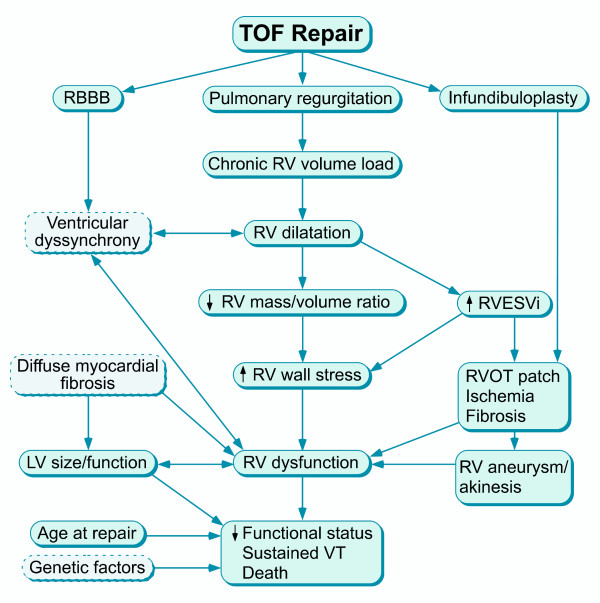
**Factors influencing right ventricular (RV) dysfunction and impaired clinical status after TOF repair**.

Human studies in repaired TOF patients using CMR provide insights regarding ventricular mechanics and clinical outcomes in this population [10,44-47]. Several authors have demonstrated a close relationship between the degree of PR and RV diastolic dimensions and stroke volume (Figure 3) [48-51]. Similar to LV function in severe chronic aortic regurgitation, once the compensatory mechanisms of the RV fail, mass-to-volume ratio decreases, end-systolic volume increases, and ejection fraction decreases (Figure 4). Kurotobi et al. demonstrated an association between an increase in RV wall stress (afterload), decreased RV ejection fraction, and symptoms in patients with repaired TOF [52]. Other factors that adversely affect RV mechanics include the spatial extent and magnitude of dyskinesis of the outflow patch (Figure 5), [19,42] RV fibrosis [42,45,53], impaired RV diastolic function,[49,54-58] and left ventricular dysfunction [10,45,59-61]. Prolonged conduction time and dyssynchrony of RV contraction likely further contribute to RV dysfunction. An older age of repair is another independent risk factor for adverse outcome in these patients [10,13,62]. Similar to the natural history of severe aortic regurgitation, in which irreversible myocardial damage follows a period of reversible ventricular dysfunction, evidence suggests that the same occurs late after TOF repair [15,63]. Figure 6 summarizes the pathophysiologic pathway that leads to ventricular dysfunction and heart failure after TOF repair.

### RV-LV Interaction After TOF Repair

The right and left ventricles function in series and, in the absence of shunts, have similar net outputs. In 1910 the French physiologist Bernheim first recognized interdependence between LV and RV function. He postulated that alterations in the size and function of the LV adversely impact the geometry and function of the RV, a phenomenon termed 'Bernheim effect' [64]. Many subsequent studies have demonstrated that alterations in the size and function of the RV lead to LV dysfunction, a phenomenon termed 'reversed Bernheim effect' [65]. Ventricular-ventricular interaction occurs because the ventricles share myofibers [66], septum, coronary blood flow, and pericardial space. The superficial spiraling layer of RV myofibers is continuous with the superficial layer of the LV whereas the deep layer of RV myofibers is continuous with that of the LV through the interventricular septum.

**Figure 7:**
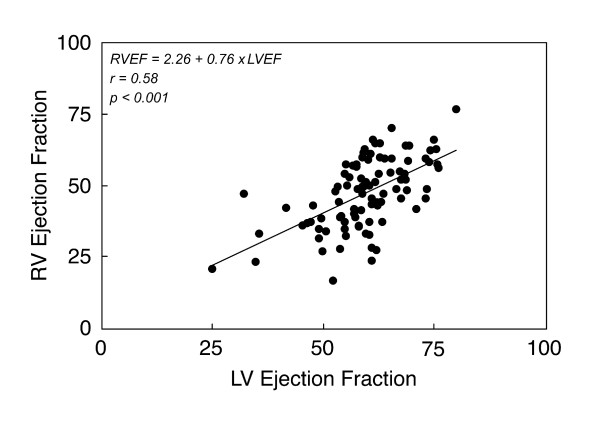
**Association between right ventricular (RV) and left ventricular (LV) ejection fraction (EF) in 100 patients with repaired TOF **[10].

The adverse impact of RV dilatation and dysfunction on LV geometry and function (both diastolic and systolic) has been demonstrated in patients with congenital (e.g., atrial septal defect, Ebstein anomaly) and acquired heart disease (e.g., pulmonary embolus, primary pulmonary hypertension). Through a complex interplay involving the shared myofibers, septum, pericardium, and coronary flow, RV volume load leads to septal shift towards the LV, leftward shift of the LV pressure-volume loop, and reduction in LV operant volumes. Initially, LV function is preserved but with progressive RV dysfunction, LV function deteriorates. Ventricular dyssynchrony, both intra- and inter-ventricular, likely contribute to adverse RV-LV interaction [60,67]. In the previously mentioned study of Kuehne et al. [19], the response of both the RV and LV to dobutamine stress was markedly blunted, suggesting that LV function is adversely influenced by impaired RV mechanics. A clinical study using CMR in patients with repaired TOF demonstrated a close linear correlation between the ejection fraction of the RV and that of the LV (Figure 7) [10].

## Natural History And Outcomes After Tof Repair

Early survival of patients with TOF is excellent with 1-2% operative mortality [68,69]. Two large cohort studies of patients with repaired TOF have shown that survival continues at ~90% during the first two decades of life [8,9]. However, mortality rate nearly triples during the third postoperative decade. Numerous studies have shown that late after TOF repair patients are at risk for exercise intolerance, heart failure, arrhythmias, and death [10,47,70-79]. In addition, pulmonary, renal, hepatic, musculoskeletal, and other non-cardiac morbidities often complicate the clinical course of these patients [47].

The common theme that has emerged from the literature on risk stratification of major adverse events (death, ventricular tachycardia, and heart failure) late after TOF repair is that there are 3 major categories of outcome predictors: 1) history (syncope, older age at repair); 2) electrophysiological markers (prolonged QRS duration, sustained ventricular tachycardia); and 3) hemodynamic sequelae of severe PR (RV dilatation, ventricular dysfunction, and regional wall motion abnormalities).

Multiple studies have addressed risk factors for sudden death and for tachyarrhythmias late after TOF repair. Gatzoulis et al. studied 793 patients from 6 centers [80]. They found that an older age at repair and QRS duration ≥180 ms were independent predictors of sudden death. Moderate or severe PR was the main hemodynamic abnormality in patients with ventricular tachycardia and sudden death, whereas tricuspid regurgitation was associated with supraventricular arrhythmias. Khairy et al., in a multicenter study of 252 patients with repaired TOF, found that a positive programmed ventricular stimulation study, history of syncope, moderate or severe pulmonary or tricuspid regurgitation, QRS duration ≥180 ms, cardiomegaly on chest radiogram (cardio-thoracic ratio ≥0.6), and multifocal premature ventricular contractions were associated with sudden death or clinical ventricular tachycardia [79]. Harrison et al. found that patients with sustained ventricular tachycardia were more likely to have RVOT aneurysms and severe PR [81]. Ghai et al., in a study that compared 12 adult patients who died late after TOF repair to 125 control patients, found that patients who died were more likely to have history of sustained ventricular tachycardia, prolonged QRS duration ≥180 ms, moderate or severe PR, and moderate or severe left ventricular dysfunction [59]. It is worth noting that in none of these studies was RV size or function quantified.

In a study that used CMR to measure PR and biventricular dimensions and function in 100 late survivors of TOF repair, RV and LV dysfunction were strongly associated with impaired clinical status, but the degree of PR was not [10]. Instead, the severity of PR was mainly related to the degree of RV enlargement, a finding that concurs with other studies that utilized CMR to assess these variables [49,82]. Knauth et al., in a follow-up study of the previous cohort, found that severe RV dilatation (end-diastolic volume Z-score ≥7 measured by CMR) and ventricular dysfunction (LV EF <55% or RV EF <45%) were independent predictors of adverse outcomes (death, sustained ventricular tachycardia, heart failure) [47]. Other univariate predictors were older age at TOF repair and QRS duration ≥180 ms. Importantly, QRS duration correlated closely with RV end-diastolic volume and, therefore, lost statistical significance in a multivariate model. Other investigators have also reported a similarly close association between QRS duration and RV size [83,84]. Myocardial fibrosis, RVOT aneurysm, and regional wall motion abnormalities have also been associated with symptoms, exercise intolerance, and arrhythmias [42,45,53,85].

## Treatment Strategies Late After Tof Repair

### Indications and Timing of Pulmonary Valve Replacement

Recommendation for PVR in patients with repaired TOF and severe PR are based on risk/benefit analysis that takes into account the natural history and pathophysiology of the disease, procedural risks, and its potential benefits.

#### Risks of Pulmonary Valve Replacement

The operative mortality for surgical PVR is low (Table 2). There is, however, continued low risk of death after PVR. Therrien et al. reported 92% survival at 5 years and 86% at 10 years in 70 adult patients after PVR [86]. Discigil et al. reported 95% survival at 5 years and 76% at 10 years in 42 patients [87]. Harrild et al. followed 98 patients after PVR (age at PVR 25 ± 13 years). Freedom from death and/or ventricular tachycardia was 80% at 5 years and 41% at 10 years, and the event incidence was 4.8 per 100 patient-years [88].

**Table 2:** Perioperative and late mortality of pulmonary valve replacement after TOF repair

Institution	Year	Number of Patients	Operative Death	Average Length of Follow-Up (years)	Late Death or transplant
SUNY, Syracuse [142]	1985	11	0	1	0
Children's Memorial Hospital, Chicago [143]	1997	49	1		
University of Toronto [101]	1997	85	1	5.8	3
Mayo Clinic [87]	2001	42	1		
Children's Hospital, Atlanta [144]	2002	100	1	4.9	1
Leiden University, The Netherlands [97]	2002	26	0	1.5	1
New England Med Center, Boston [105]	2003	36	0	5	1
University of Zurich, Switzerland [100]	2005	39	0	1.25	0
Multicenter, The Netherlands [145]	2006	158	0	4.2	2
University of Toronto [99]	2007	82	0	8.8	2
University Medical Center, Rotterdam [14]	2008	17	0	6.4	0
International Society of Congenital Heart Disease [107]	2008	93	0	3	2
Great Ormond Street, London[94]	2008	71	0	1	0
Emory University [146]	2009	107	3		
Children's Hospital Boston [88]	2009	77	0	2.8	6
Children's Hospital, Atlanta [147]	2010	42	0	2.2	0

		1035	0.68%		2.2%

When considering the risks associated with PVR, the risk of valve failure should also be considered. All valves inserted in the pulmonary position have a limited life expectancy, with wide variations in rates of freedom from valve failure and reoperation, depending of the type of valve and patient age. Calderone et al. reported freedom from reoperation in 81% of patients at 5 years, 58% at 10 years, and 41% at 15 years [89]. These authors, as well as many others, have shown that young age at pulmonary valve placement is associated with a higher rate of valve failure and early reoperation.

The advent of transcatheter pulmonary valve implantation [90,91], however, may provide a new non-surgical option for the treatment of failed bioprosthetic pulmonary valve. Although the currently available catheter-delivered, stent-mounted valves are limited by the size and geometry of the RVOT to mostly patients with RV-to-pulmonary artery conduits, future developments will likely expand the clinical application of this technique to patients with dilated RVOT. Even with currently available technology, catheter-based pulmonary valve implantation can be performed inside a failing bioprosthetic valve or conduit [92]. Further development of this technology may reduce the need for reoperation after pulmonary valve implantation and will likely lower the threshold for restoring pulmonary valve competency in patients with chronic PR.

#### Benefits of Pulmonary Valve Replacement

There is strong evidence that PVR is highly effective in eliminating or greatly reducing PR [93-95]. Multiple studies published during the past decade have painted a consistent picture regarding the clinical response and the mechanical adaptation of the right and left ventricles to PVR [96]. Table 3 summarizes findings from 10 representative studies. Clinically, many patients report less cardiac symptoms after PVR and several studies have reported a significant improvement in NYHA functional class [15,93-95,97-99]. Within approximately one year after PVR RV end-diastolic and end-systolic volumes decrease by 30-40% as compared with their preoperative values. On average, global RV systolic function (measured as ejection fraction) remains unchanged. LV end-diastolic volume increases slightly whereas global LV systolic function remains unchanged [15,16,63,86,87,95,100-103]. The degree of tricuspid valve regurgitation (evaluated by Doppler echocardiography) tends to improve with or without concomitant tricuspid valve surgery. The percent of patients with at least moderate tricuspid regurgitation decreased from 25% before PVR to 5% after surgery in the study of Buechel et al. [16], from 24% to 0% in the study of Therrien et al. [15], and from 13% to 2% in the study of Geva et al [95].

**Table 3:** Effects of pulmonary valve replacement on ventricular mechanics, QRS duration, peak oxygen consumption, and functional class

	PR (%)		RVEDVi (ml/m^2^)		RVESVi (ml/m^2^)		RV EF (%)		LVEDVi (ml/m^2^)		LV EF (%)		QRS duration (ms)		Peak O_2 _ consumption (ml/kg/min)		NYHA class	
	**Before**	**After**	**Before**	**After**	**Before**	**After**	**Before**	**After**	**Before**	**After**	**Before**	**After**	**Before**	**After**	**Before**	**After**	**Before**	**After**

Vliegen et al. [97] N = 26 Age: 29 ± 9 years	46 ± 10	4 ± 8	167 ± 40	114 ± 35	99 ± 36	66 ± 35	42 ± 10	42 ± 11	86 ± 29	87 ± 17							2.0 ± 0.6	1.3 ± 0.5
Therrien et al. [15] N = 17 Age: 32 years			163 ± 34	107 ± 26	109 ± 27	69 ± 22	32 ± 7	34 ± 10									2.0 ± 1.0	1.4 ± 0.5
van Straten et al. [148] N = 16 Age: 29 years	48 ± 10	3 ± 5	164 ± 43	113 ± 26	94 ± 33	61 ± 18	44 ± 8	47 ± 12										
Doughan et al. [84] N = 21 Age: 34 ± 9 years													153 ± 34	142 ± 29				
Buechel et al. [16] N = 20 Age: 14 ± 3 years	49 ± 14	9 ± 8	190 ± 33	109 ± 26	102 ± 27	58 ± 16	47 ± 7	45 ± 9	77 ± 10	84 ± 12	53 ± 6	56 ± 7	150 ± 18	148 ± 17				
Henkens et al. [98] N = 27 Age: 31 ± 8 years	48 ± 11		166	100	98	58	42 ± 10	43 ± 10	89 ± 31	87 ± 18	56 ± 12	55 ± 9					2.0 ± 0.6	1.3 ± 0.3
Oosterhof et al. [93] N = 71 Age: 29 years	44 ± 13	5 ± 9	171 ± 44	119 ± 34	102 ± 38	70 ± 29	42 ± 10	43 ± 10	85 ± 22	94 ± 20	52 ± 9	53 ± 8	155 ± 29	144 ± 29			53% grade ≥II	11% grade ≥II
Gengsakul et al. [99] N = 82 Age: 28 ± 13 years													164 ± 21	168 ± 21			54% grade ≥II	13% grade ≥II
Frigiola et al. [94] N = 71 Age 22 ± 11 years	41 ± 9	5 ± 7	142 ± 43	91 ± 18	73 ± 33	43 ± 14	51 ± 10	54 ± 7	66 ± 12	73 ± 13	61 ± 8	64 ± 7			25 ± 10	25 ± 9	2.0	1.0
Geva et al. [95] N = 64 Age: 21 years	49 ± 11	5 ± 9	201 ± 37	123 ± 25	107 ± 29	68 ± 24	47 ± 8	45 ± 9	89 ± 15	94 ± 17	58 ± 8	57 ± 7	154 (82-200)	150 (80-202)	26.5 (8-47)	27 (10-48)	47% grade ≥II	8% grade ≥II

The data regarding the effects of PVR on QRS duration, arrhythmia propensity, and objective exercise parameters is inconsistent. Some investigators have reported modest improvements in one or more of these categories whereas others have shown no significant change from pre- to post-PVR. Therrien et al. reported that the incidence of ventricular tachycardia was lower after PVR (9% post operatively versus 23% preoperatively) and that the rate of increase in QRS duration had stabilized after the procedure [86]. In contrast, Harrild et al. and Gengsakul et al. found no significant improvement in the frequency of arrhythmias after PVR [88,99]. Similarly conflicting results have been reported regarding decrease in QRS duration from pre- to post-PVR [16,84,88,95,104]. Finally, whereas some investigators have reported that certain indices of exercise tolerance improve [94,102,105], others have not found such improvement [95,106].

#### Rationale and Timing of Pulmonary Valve Replacement

**Figure 8:**
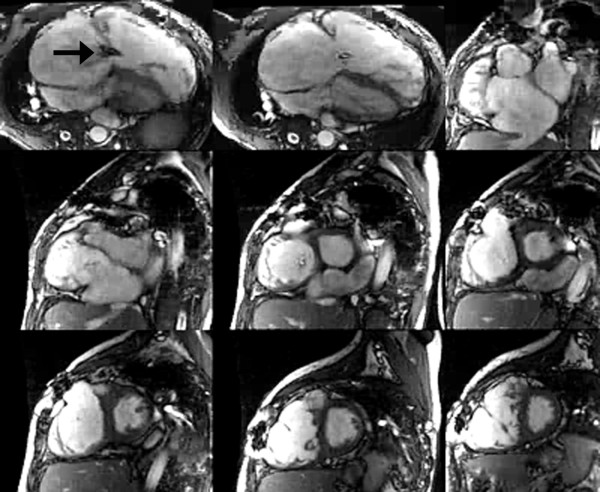
**CMR imaging in a 37 year-old patient with repaired TOF, severe pulmonary regurgitation, moderate tricuspid regurgitation (arrow), and severe right ventricular dilatation (end-diastolic volume index 386 ml/m**^**2**^**) and dysfunction (ejection fraction 15%)**. This patient who also exhibited severe heart failure symptoms had only modest decrease in RV size and no improvement in RV function after pulmonary valve replacement.

Although conclusive confirmation that PVR improves survival late after TOF repair is still lacking, a growing body of evidence has emerged during the last decade supporting the rationale for the procedure. There is strong evidence that without intervention severe PR in repaired TOF leads to severe RV dilatation and dysfunction, tricuspid valve regurgitation, LV dysfunction, tachyarrhythmias, diminished exercise tolerance, heart failure symptoms, and death (Additional file 1) [8-10,59,80]. As discussed above, PVR leads to elimination or marked reduction of PR, improved biventricular mechanics, improvement in tricuspid regurgitation, and symptomatic improvement. For many years most centers have referred patients with severe chronic PR for PVR based on overt symptoms, such as progressive exercise intolerance, heart failure symptoms, syncope, or ventricular tachycardia [17,63,88,107]. Recent evidence clearly demonstrates that relying on symptoms as the major criteria for PVR results in patients receiving a pulmonary valve when their RV is markedly dilated (mean RV end-diastolic volume 201 ± 37 ml/m^2 ^in one study [95]), and RV and/or LV dysfunction is present (Figure 8). Several studies have identified pre-PVR threshold values of RV end-diastolic and end-systolic volumes that are associated with postoperative normalization of RV size. Therrien et al. reported that RV size did not return to normal in any of the 7 patients whose pre-operative end-diastolic volume index was >170 ml/m^2^, whereas RV size normalized in 9 of 10 patients with pre-operative end-diastolic volume ≤170 ml/m^2 ^[15]. Oosterhof et al. identified RV end-diastolic volume <160 ml/m^2 ^and end-systolic volume <82 ml/m^2 ^as associated with normal postoperative RV size [93]. Buechel et al. identified RV end-diastolic volume <150 ml/m^2 ^as the threshold value below which RV size returns to the normal range after PVR [16]. Frigiola et al. have adopted an institutional policy of recommending PVR in asymptomatic patients based on RV dilatation (RV/LV ratio >2) and abnormal exercise test results [94]. With an average preoperative RV end-diastolic volume of 142 ± 43 ml/m^2^, RV end-systolic volume of 91 ± 18 ml/m^2^, and RV ejection fraction 47 ± 8%, RV size and function was, on average, within normal limits one year after PVR. The authors, therefore, recommend PVR before RV end-diastolic volume exceeds 150 ml/m^2^. We analyzed pre-PVR predictors of normal post-PVR RV size (end-diastolic volume index ≤114 ml/m^2^) and function (ejection fraction ≥48%) in 64 patients with severe chronic PR [95]. Independent predictors of normal RV size and function were pre-operative RV end-systolic volume index <90 ml/m^2 ^and QRS duration <140 ms.

Thus, the timing and indications for PVR after TOF repair must balance the benefits of elimination of RV volume load before irreversible dysfunction occurs and the disadvantages of a premature surgical or transcatheter procedure. As with any other medical procedure, the risks and benefits of the procedure must be weighed on a case-by-case basis, taking into account not only cardiac risk factors but also the risks associated with non-cardiac morbidities. With the above considerations in mind, the following summarizes the author's current recommendations for PVR in patients with repaired TOF or similar physiology. These recommendations will undoubtedly continue to evolve as new information becomes available and as valve technology and implantation methods continue to improve.

#### Indications for Pulmonary Valve Replacement

Indications for PVR in patients with repaired TOF or similar physiology with moderate or severe pulmonary regurgitation (regurgitation fraction ≥25%):

##### I. Asymptomatic patient with two or more of the following criteria

a. RV end-diastolic volume index >150 ml/m^2 ^or Z-score >4. In patients whose body surface area falls outside published normal data: RV/LV end-diastolic volume ratio >2

b. RV end-systolic volume index >80 ml/m^2^

c. RV ejection fraction <47%

d. LV ejection fraction <55%

e. Large RVOT aneurysm

f. QRS duration >140 ms

g. Sustained tachyarrhythmia related to right heart volume load

h. Other hemodynamically significant abnormalities:

○ RVOT obstruction with RV systolic pressure ≥2/3 systemic

○ Severe branch pulmonary artery stenosis (<30% flow to affected lung) not amenable to transcatheter therapy

○ ≥ Moderate tricuspid regurgitation

○ Left-to-right shunt from residual atrial or ventricular septal defects with pulmonary-to-systemic flow ratio ≥ 1.5

○ Severe aortic regurgitation

○ Severe aortic dilatation (diameter ≥5 cm)

##### II. Symptomatic Patients

Symptoms and signs attributable to severe RV volume load documented by CMR or alternative imaging modality, fulfilling ≥1 of the quantitative criteria detailed above. Examples of symptoms and signs include

a. Exercise intolerance not explained by extra-cardiac causes (e.g., lung disease, musculoskeletal anomalies, genetic anomalies, obesity), with documentation by exercise testing with metabolic cart (≤70% predicted peak VO_2 _for age and gender not explained by chronotropic incompetence)

b. Signs and symptoms of heart failure (e.g., dyspnea with mild effort or at rest not explained by extra-cardiac causes, peripheral edema)

c. Syncope attributable to arrhythmia

##### III. Special considerations

a. Due to higher risk of adverse clinical outcomes in patients who underwent TOF repair at age ≥3 years [10], PVR may be considered if fulfill ≥1 of the quantitative criteria in section I

b. Women with severe PR and RV dilatation and/or dysfunction may be at risk for pregnancy-related complications [108]. Although no evidence is available to support benefit from pre-pregnancy PVR, the procedure may be considered if fulfilling ≥1 of the quantitative criteria in section I

## Role of CMR

Because it is not limited by acoustic window, not associated with exposure to ionizing radiation, and is noninvasive, CMR is ideally suited for longitudinal follow-up in patients with repaired TOF [109]. Since it provides accurate quantitative information on biventricular size and function, blood flow measurements, myocardial viability, and cardiovascular anatomy, in many centers CMR has become the preferred method of noninvasive imaging in patients with repaired TOF [10,45,50,54,97,110-113]. At Children's Hospital Boston, postoperative TOF is the most common diagnosis in patients referred for CMR, accounting for ~23% of patients. However, comprehensive assessment of the patient with repaired TOF requires integration of information from clinical assessment (detailed history and physical examination) and laboratory investigations (ECG, Holter monitor, exercise test, and echocardiography). Computed tomography and radionuclear studies can be used when CMR is contraindicated or not available. Cardiac catheterization (with or without electrophysiological testing) is indicated in selected patients [17,95,114].

### Goals of CMR

The goals of CMR in patients with repaired TOF include:

• Quantitative assessment of left and right ventricular volumes, mass, stroke volumes, and ejection fraction.

• Evaluation of regional wall motion abnormalities.

• Imaging the anatomy of the right ventricular outflow tract, pulmonary arteries, aorta, and aorto-pulmonary collaterals.

• Quantification of PR, tricuspid regurgitation, cardiac output, and pulmonary-to-systemic flow ratio.

• Assessment of myocardial viability with particular attention to scar tissue in the ventricular myocardium aside from sites of previous surgery (e.g., ventricular septal defect and RVOT patches).

### Study Protocol

• The importance of careful attention to details of patient preparation and placement in the scanner cannot be overemphasized. Optimal placement of ECG leads is paramount to quality gating. A peripheral intravenous cannula for injection of gadolinium-based contrast is placed in the following circumstances:

1. First CMR examination

2. >3 years since last late gadolinium enhancement (LGE) evaluation

3. Deterioration in clinical status

4. Regional or global ventricular function has worsened

Exceptions are made in young patients when placement of an intravenous cannula might result in loss of patient cooperation.

#### Imaging protocol

**Figure 9:**
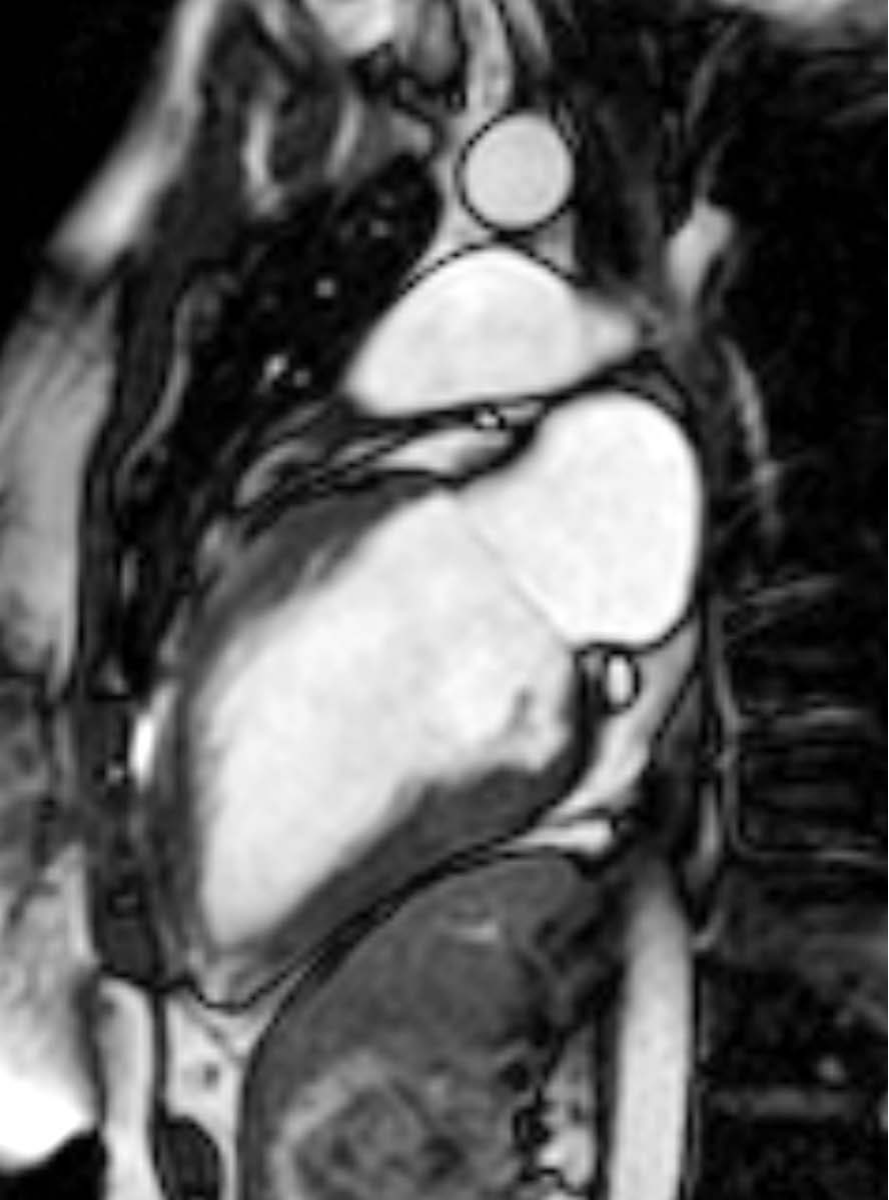
**Evaluation of ventricular size and function by ECG-gated cine SSFP MR in repaired TOF: Left ventricular 2-chamber (vertical long-axis) plane**.

• **Localizing images**: ECG-gated steady-state free precession (SSFP) localizing imaging in the axial, coronal, and sagittal planes followed by real-time interactive sequences for identification of key imaging planes and structures targeted for additional sequences (e.g., ventricular long- and short-axis planes, short-axis of the proximal MPA for subsequent measurements of PR).

• **ECG-triggered, breath-hold cine SSFP **in the following planes:

▪ **LV 2-chamber **(Figure 9)

**Figure 10:**
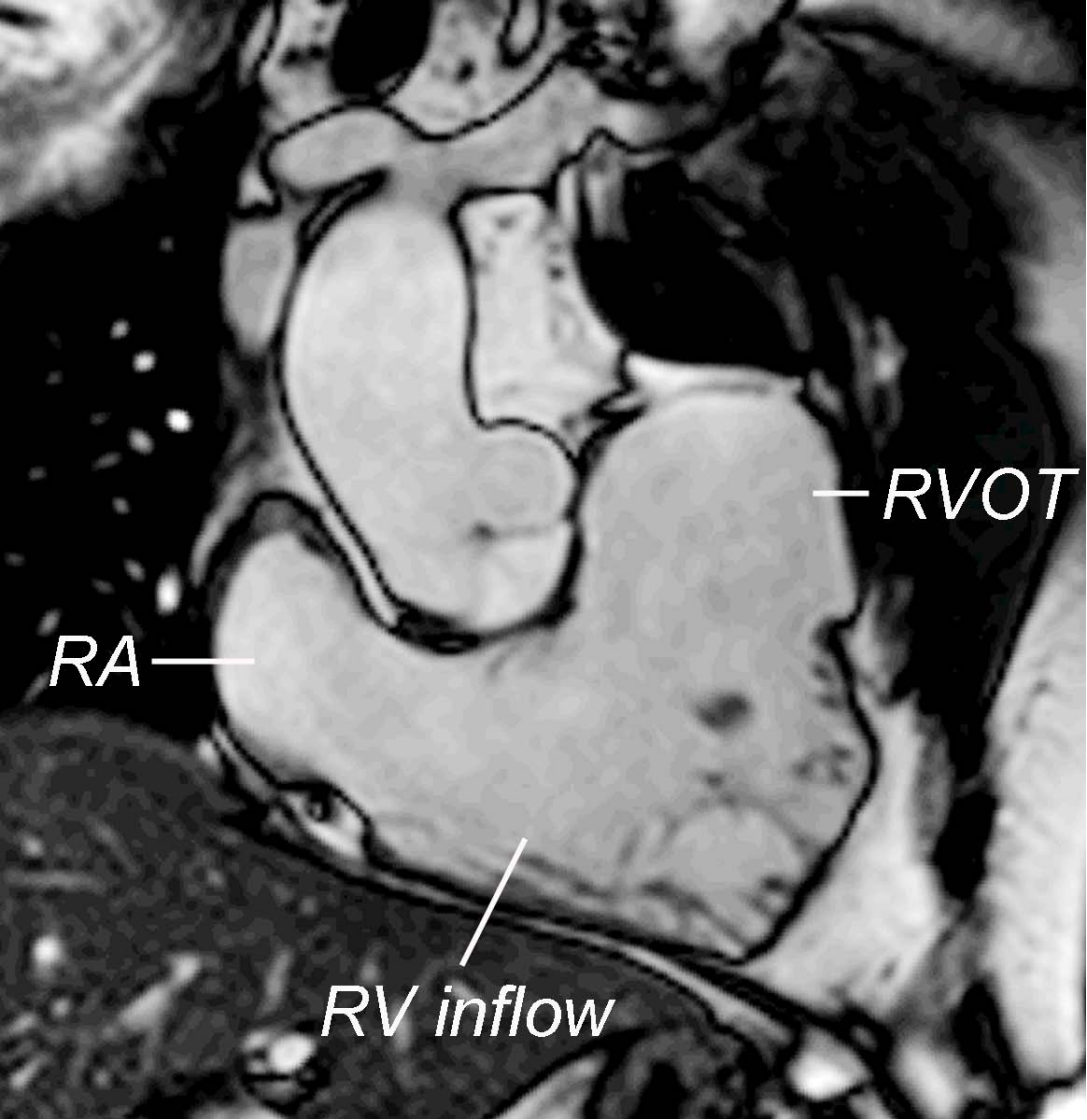
**Evaluation of ventricular size and function by ECG-gated cine SSFP MR in repaired TOF: Right ventricular 2-chamber (vertical long-axis) plane**. RA = right atrium; RV = right ventricle; RVOT = right ventricular outflow tract.

▪ **RV 2-chamber **(Figure 10)

**Figure 11:**
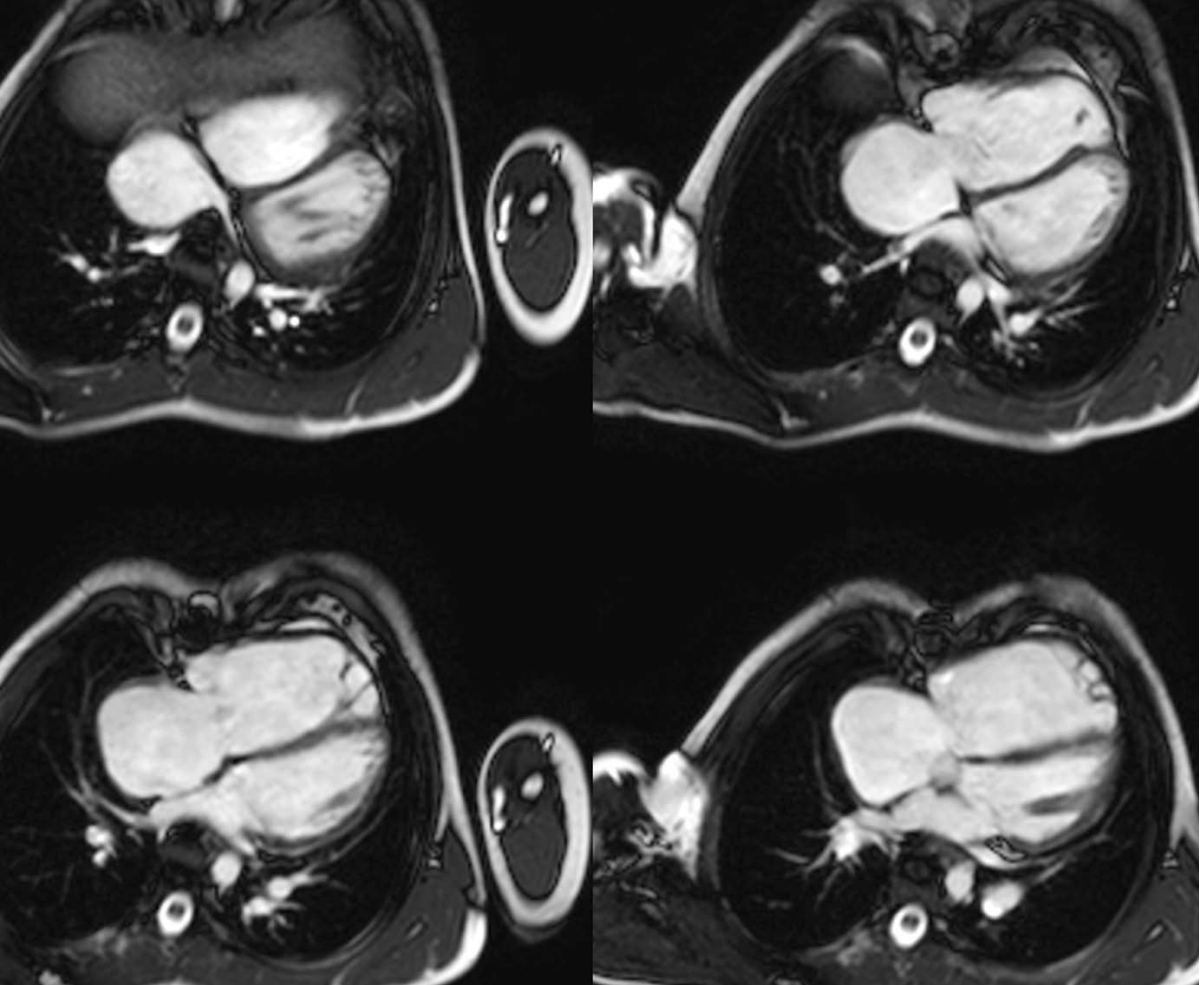
**Evaluation of biventricular size and function by ECG-gated cine SSFP MR in repaired TOF: 4-chamber (horizontal long-axis) plane**.

▪ **4-chamber **(4 slices) (Figure 11)

**Figure 12:**
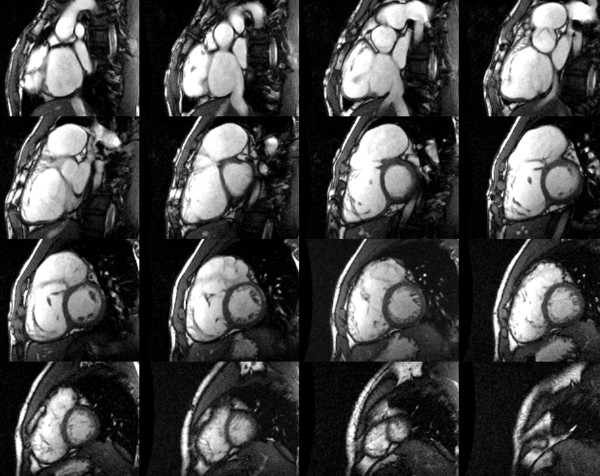
**Evaluation of biventricular size and function by ECG-gated cine SSFP MR in repaired TOF: Ventricular short-axis. Note that 16 short-axis slices were required to fully cover the markedly dilated RV in this patient**.

▪ **Ventricular short-axis **(Figure 12). The latter is achieved by prescribing 12-14 equidistant slices (slice thickness 6-8 mm; inter-slice space 0-2 mm) covering the entire length of both ventricles. Particular attention is given to inclusion of the base of the RV and LV at end-diastole with addition of extra slices as needed for complete coverage.

**Figure 13:**
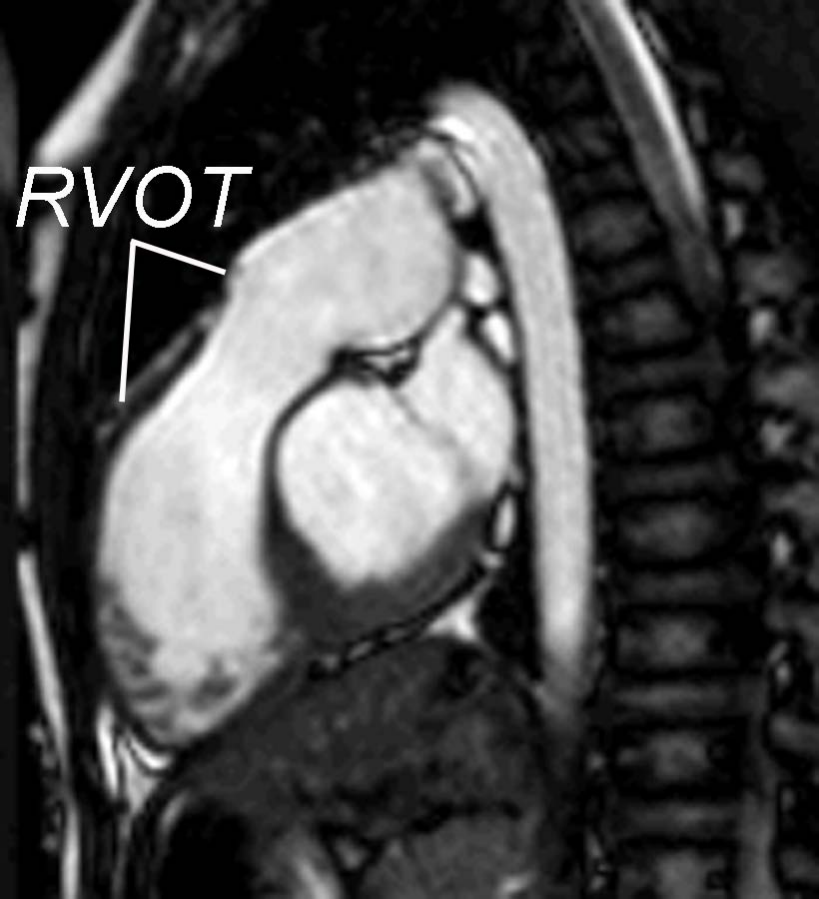
**Evaluation of the right ventricular outflow tract (RVOT) long-axis by ECG-gated cine SSFP MR**. Along with the RV 2-chamber plane (Figure 10), this view demonstrates patency of the RVOT and main pulmonary artery, presence or absence of pulmonary valve tissue, and wall motion abnormalities.

▪ Oblique sagittal parallel to the **RVOT and proximal MPA **(Figure 13, Additional file 2).

▪ Parallel to the **left ventricular outflow **(LV 3-chamber view).

▪ **Axial plane **for imaging of the **outflow tracts and branch pulmonary arteries **(all first studies, optional thereafter).

Representative imaging parameters: echo time 1.7 ms; repetition time 3.3 ms; flip angle 60°; sensitivity encoding (SENSE) acceleration factor 2; field of view 260 mm; matrix 160 × 160 reconstructed to 256 × 256; voxel size 1.6 × 1.8 × 6-8 mm reconstructed to 1.0 × 1.0 × 6-8 mm; 30 reconstructed images per cardiac cycle.

**Figure 14:**
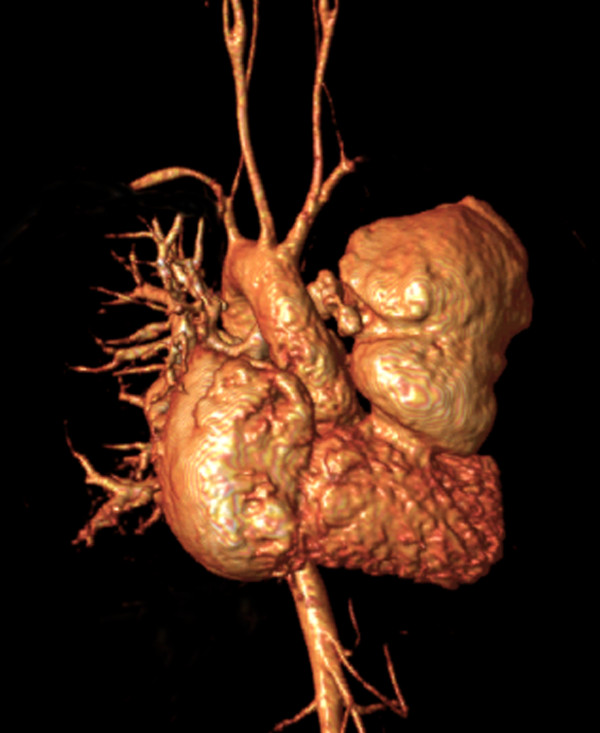
**Gadolinium-enhanced 3-dimensional magnetic resonance angiography in a patient with repair TOF and a giant aneurysm of the outflow patch**.

**Figure 15:**
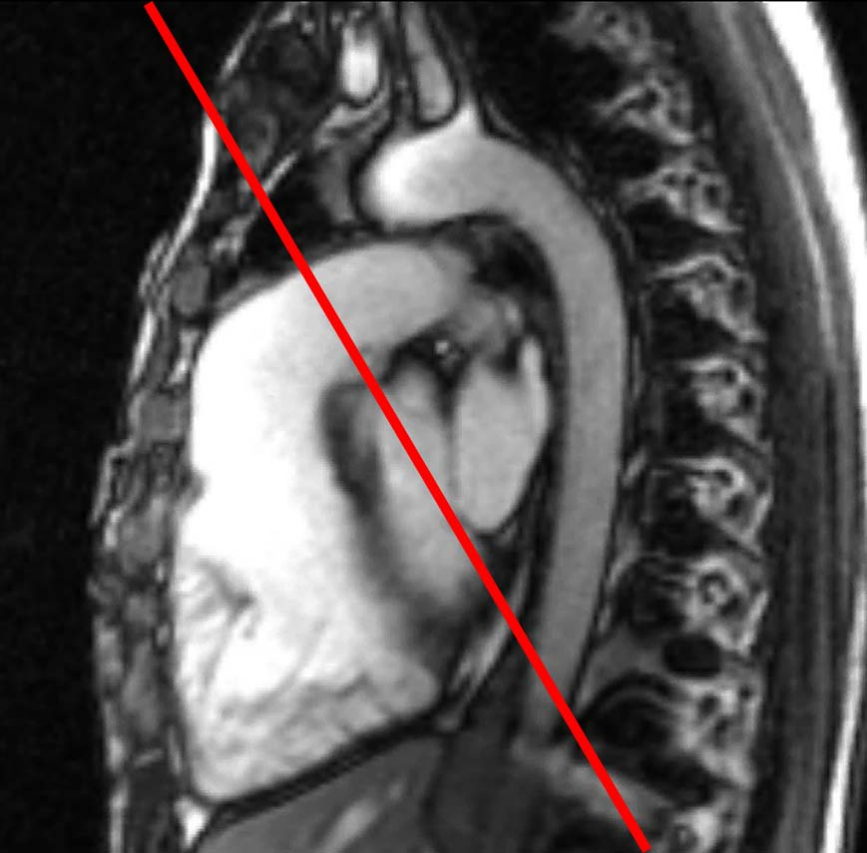
**Evaluation of pulmonary regurgitation (PR) by ECG-gated cine phase contrast MR: The imaging plane is placed perpendicular to the long-axis of the main pulmonary artery (MPA)**.

**Figure 16:**
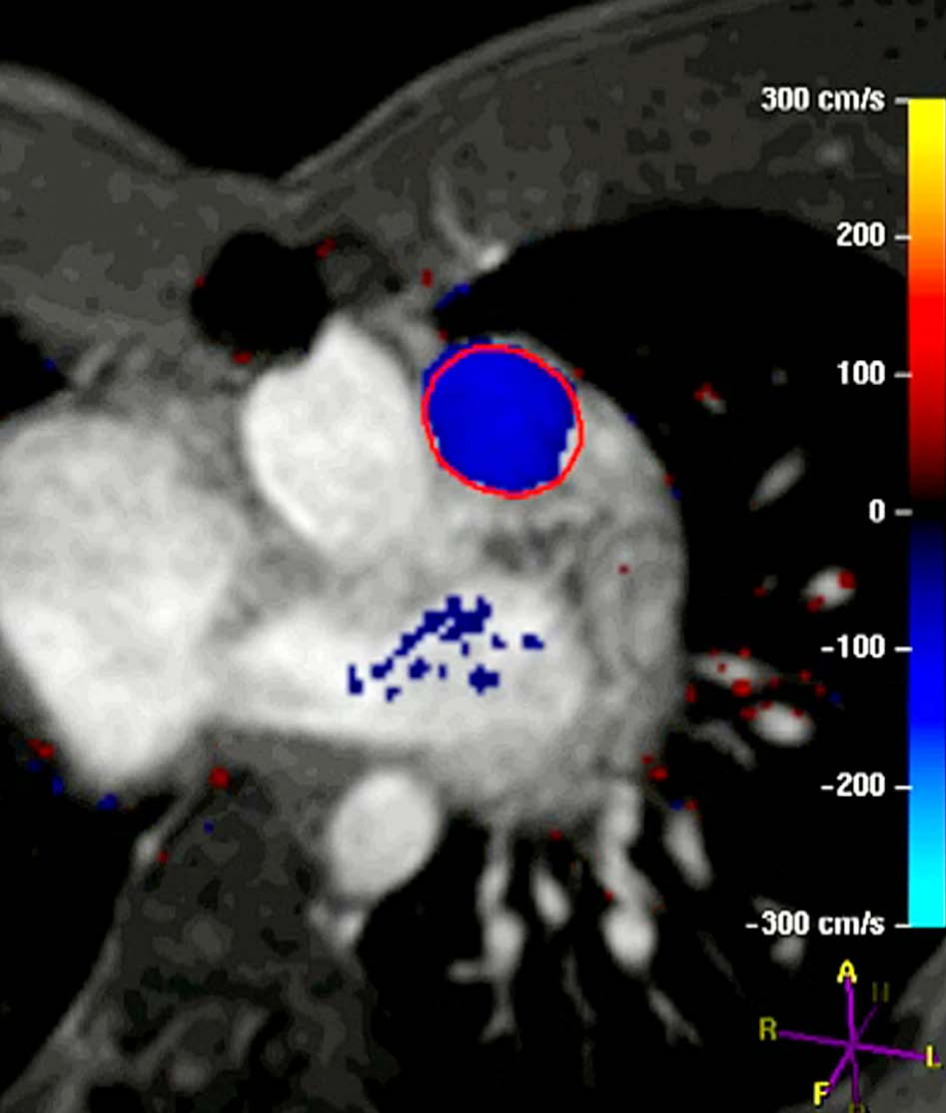
**Evaluation of pulmonary regurgitation (PR) by ECG-gated cine phase contrast MR: Color-coded flow map of the main pulmonary artery with the region of interest contour shown at peak systole**.

**Figure 17:**
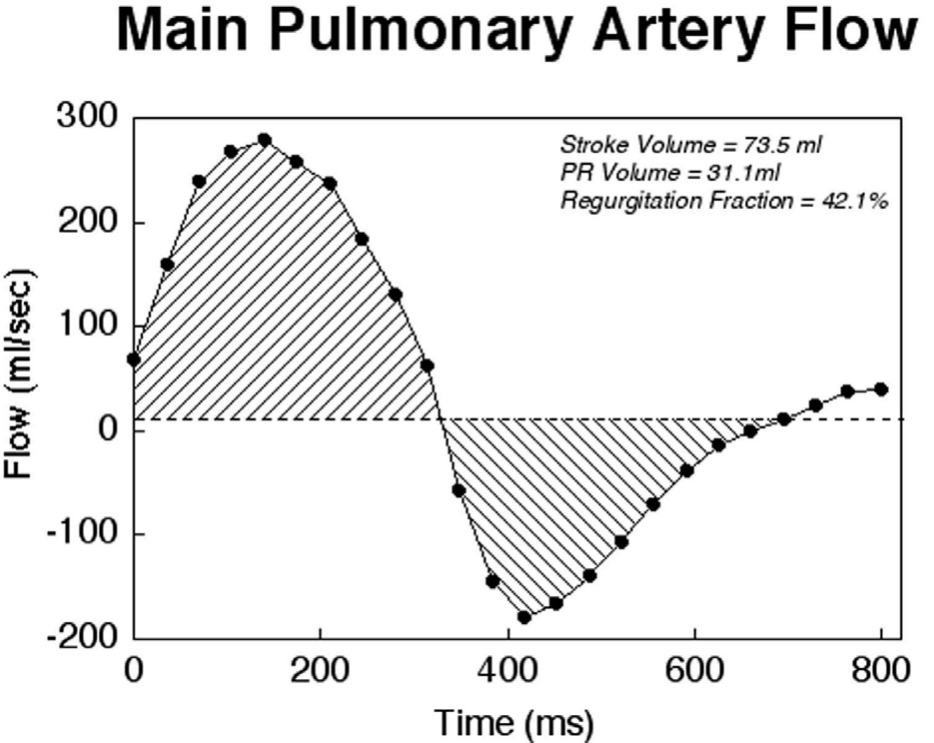
**Evaluation of pulmonary regurgitation (PR) by ECG-gated cine phase contrast MR: MPA flow rate (Y-axis) versus time (X-axis)**. Flow above the baseline represents antegrade flow and flow below the baseline represents retrograde (regurgitation) flow.

**Figure 18:**
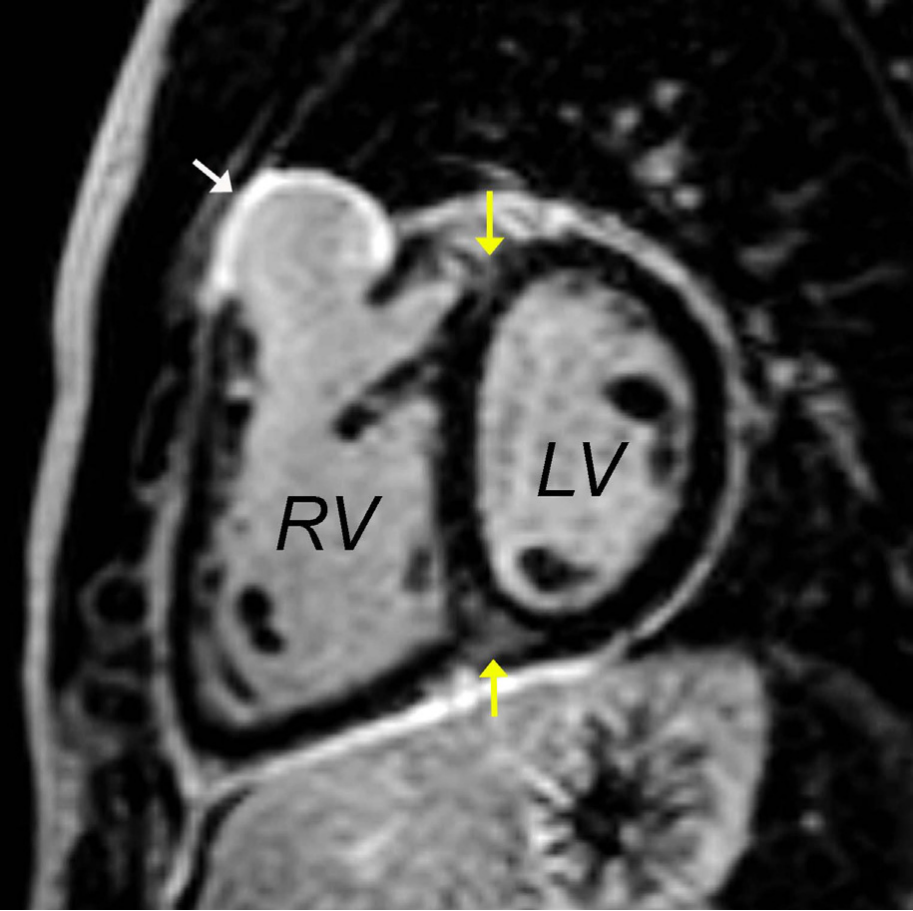
**Late gadolinium enhancement imaging in the ventricular short-axis showing intense late hyperenhancement in the RVOT (white arrow) and weak hyperenhancement in the superior and inferior junctions between the interventricular septum and the free wall (yellow arrows). The former represents scar tissue and is associated with regional wall motion abnormalities**. The latter is a commonly observed finding in patients with repaired TOF and its clinical importance is uncertain [42,85].

• **Magnetic resonance angiogram (MRA)**: Non-gated, breath-hold gadolinium-enhanced (0.2 mmol/kg gadopentetate dimeglumine) 3-dimensional MRA (all first studies, optional thereafter) (Figure 14). Representative imaging parameters: echo time 1.5 ms; repetition time 4.5 ms; flip angle 40°; voxel size 0.95 × 1.06 × 2.4 mm reconstructed to 0.68 × 0.69 × 1.2 mm; number of acquisitions 2; SENSE acceleration factor 2; and acquisition time 20 s/acquisition.

• **Flow measurements**: ECG-triggered, breathe-through cine phase contrast flow measurements in the **MPA **(Figures 15, 16, 17, Additional file 3), **aorta**, and **atrioventricular valves**. Representative imaging parameters: echo time 3.7 ms; repetition time 5.9 ms; flip angle 15°; SENSE factor 2; field of view 300 mm; matrix 192 × 192; voxel size 1.56 × 1.56 × 6.0 mm reconstructed to 1.17 × 1.17 × 6.0 mm; 40 reconstructed images per cardiac cycle.

• **Late gadolinium enhancement (LGE)**: ECG-triggered, breath-hold, phase sensitive LGE imaging performed 10-20 minutes after contrast administration in the following planes: ventricular short-axis (Figure 18), LV 2-chamber, LV 3-chamber, RV 2-chamber, and 4-chamber. Areas suspected of LGE (e.g., the thin walled RVOT free wall) are imaged in orthogonal planes and with phase direction swapped to facilitate recognition of artifacts.

In addition to the above, the following imaging sequences are performed on a case-by-case basis:

• ECG-triggered, breath-hold cine SSFP in the **short-axis of the aortic root and ascending aorta **(in patients with dilated aortic root and ascending aorta).

• ECG-triggered, breath-hold turbo (fast) spin echo sequence with blood suppression for imaging of the **outflow tracts and branch pulmonary arteries **in patients with image artifacts from metallic implants.

• ECG-triggered, breathe-through cine phase contrast flow measurements in the **branch pulmonary arteries **(when branch pulmonary artery stenosis is identified).

• ECG-triggered, respiratory navigated, free breathing 3-dimensional isotropic SSFP for evaluation of the **coronary arteries **or as a substitute for contrast magnetic resonance angiography.

In a cooperative patient the above protocol is typically completed in 65-70 minutes.

### Report Template

The importance of a structured report with an organized, comprehensive structure that addresses all key elements pertinent to clinical decision-making cannot be overemphasized. Key data reporting elements include:

• Anatomy of the RVOT and main and branch pulmonary arteries with emphasis on obstruction and/or dilatation or aneurysm formation.

• Biventricular size and function (global and regional).

• Vessel dimensions: aortic root, ascending aorta, MPA, right and left pulmonary arteries; if abnormal (e.g., tricuspid regurgitation), diameters of the atrioventricular valve.

• Flow measurements:

▪ Ascending aorta, MPA, right and left pulmonary arteries

▪ Pulmonary valve regurgitation

▪ Other valve regurgitation

• Late gadolinium enhancement: presence, location, and extent.

• Associated anomalies: systemic and pulmonary veins, aortic arch sidedness and branching order.

An example of CMR report in a hypothetical patient with repaired TOF is available in Additional File 4.

### Offline analysis

**Figure 19:**
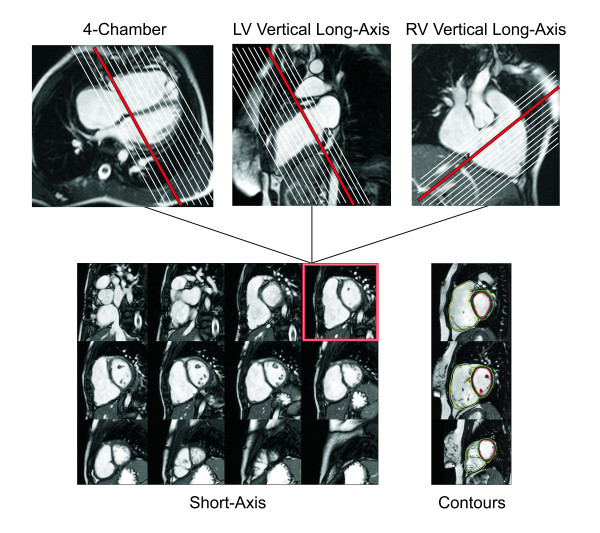
**CMR assessment of biventricular volumes and mass in repaired TOF**. Cross-referencing between ventricular long- and short-axis imaging planes aids determining inclusion of basal slices in the ventricular volume analysis. When an operator selects a frame on the short-axis grid, that location is highlighted on the linked horizontal and vertical long axis images, allowing the operator to determine the location of the slab relative to the atrioventricular valves. Right lower panel: Examples of contour drawings on the left and right ventricular endocardial and epicardial boundaries at the base, mid-ventricular, and apical levels.

Quantification of right and left ventricular size and function and blood flow is performed using dedicated software available either from the manufacturers of MRI equipment or from third party vendors. Measurements of biventricular diastolic and systolic volumes and mass are performed on ECG-gated cine SSFP images, which provide a high contrast between the blood pool (T2/T1 = 360/1200 = 0.3) and the myocardium (T2/T1 = 75/880 = 0.085) [115]. Accurate determination of ventricular volume requires clear depiction of the blood-myocardial boundary. Adjustments of the image brightness and contrast on the computer screen can facilitate visualization of that boundary. By tracing the blood-endocardium boundary, the slice's blood pool volume is calculated as the product of its cross-sectional area and thickness (which is prescribed by the operator) (Figure 19). The left ventricular papillary muscles and the major trabeculations of the RV (e.g., septal band) are excluded from the blood pool and are considered part of the myocardium (Figure 19) [46,116]. Ventricular volume is then determined by summation of the volumes of all slices. The process can be repeated for each frame in the cardiac cycle to obtain a continuous time-volume loop or may be performed only on end-diastolic (maximal area) and end-systolic (minimal area) frames to calculate diastolic and systolic volumes. From this data one can calculate left and right ventricular stroke volumes and ejection fractions. Since the patient's heart rate at the time of image acquisition is known, one can calculate left and right ventricular outputs. Ventricular mass is calculated by tracing the epicardial borders and calculating the epicardial volume, subtracting the endocardial volume, and multiplying the resultant muscle volume by the specific gravity of the myocardium (1.05 g/mm^3^).

Several approaches to measurements of biventricular size and function have been reported. In general, these can be divided into 2 broad categories: 1) methods that rely on summation of discs (Simpson's principle) [117]; and 2) methods that rely on modeling the chamber or extrapolation of sparse data [118-120]. In the first category each of the slices covering the ventricles is contoured at least once at end-diastole (largest volume) and at end-systole (smallest volume), requiring tracing 72 contours for biventricular volumes and mass. Some groups have advocated the use of images obtained in axial or oblique long-axis planes [121,122]. The major advantage of this approach, as compared with analysis based on short-axis images, is the ease of determining the planes of the mitral, tricuspid, and pulmonary valves. This advantage is likely responsible for the slightly higher reproducibility of measurements using older software [122]. However, this approach limits evaluation of ventricular mass because the epicardial and endocardial borders of the diaphragmatic wall are not clearly defined. Recent development of techniques that incorporate cross-references between long- and short-axis images has greatly reduced the difficulty in determining valve plane on short-axis images (Figure 19) [123]. Moreover, most reports on normal values as well as the majority of the literature on ventricular size and function in repaired TOF and other congenital and acquired anomalies is based on analysis of short-axis images [116,124,125].

In the second category either a formula based on a geometrical model or extrapolation from sparse data are used to generate ventricular volumes [118-120]. The major advantage of this approach is shorter analysis time but it is disadvantaged by reduced accuracy.

**Figure 20:**
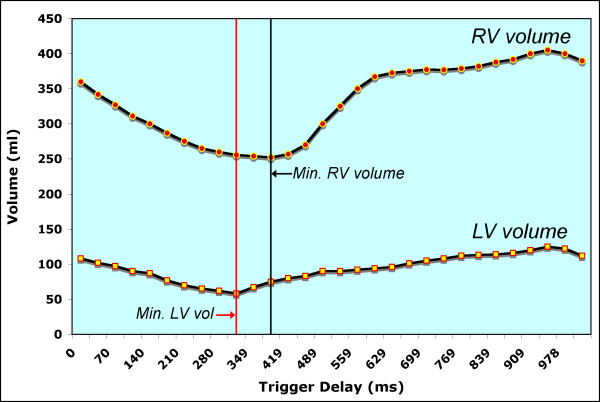
**Plot of left and right ventricular volumes versus time throughout the cardiac cycle in a patient with repaired TOF**. Note that minimal RV volume occurs 70 ms (2 cardiac phases) after minimal LV volume.

In our center ventricular volumes and mass are measured from short-axis cine SSFP images [46]. Cross-referencing the short-axis images with left and right ventricular 2-chamber (vertical long-axis) and 4-chamber (horizontal long-axis) cine SSFP facilitates accurate determination of the atrioventricular and semilunar valves planes during systole and diastole [123]. In patients with repaired TOF particular attention should be paid when determining the end-diastolic and end-systolic phases of each ventricle. Given that conduction delay is nearly universal in this population, peak RV contraction typically lags after that of the LV by 1-3 cardiac phases (Figure 20). To optimize interstudy reproducibility in patients followed longitudinally, contours should be compared side-by-side with those from previous studies. Saving the contour files along with previous studies facilitates this comparison. On average, total analysis time is ~30 minutes and decreases with operator experience [123].

The technique for measuring blood flow is well established [109]. PR fraction is calculated as retrograde flow volume divided by antegrade flow volume in the proximal MPA using ECG-gated, free-breathing cine phase contrast sequence obtained in the short-axis of the proximal MPA (Figure 15 and 16) [46]. The operator should adjust the imaging plane to avoid impingement on the MPA by metallic artifacts from sternal wires and implants. In addition to the PR fraction, both the antegrade and retrograde flow volumes should be reported [126]. In the absence of a residual shunt, the net flow in the MPA and ascending aorta should be nearly identical. Similarly, in the absence of important tricuspid, mitral or aortic valve regurgitation or shunt, left and right ventricular stroke volume differential is primarily affected by PR and calculation of PR fraction by the 2 methods should be similar. In 15-20% of patients with repaired TOF, however, residual shunt(s) and tricuspid and/or aortic valve regurgitation are present, thus limiting the use of these comparisons [95].

### Reproducibility of CMR Measurements

Most studies on the reproducibility of volumetric measurements by CMR have focused on the LV and those that included RV measurements were based on healthy volunteers or patients with acquired heart disease [124,127]. Few studies have addressed reproducibility of volumetric measurements by CMR in patients with repaired TOF [119,123,128]. Extrapolation of data from healthy volunteers or from adults with acquired heart disease to this group of patients may not be applicable because the RV is typically dilated and hypertrophied, the apical trabeculations are extensive, chamber geometry is abnormal with a prominent subtricuspid protrusion (the "shoulder" of the RV), the outflow patch is dyskinetic, the plane of the pulmonary valve can be unclear, and metallic artifacts from sternal wires or other implants can obscure parts of the chamber.

Mooij et al. analyzed the intra- and inter-observer variability of RV and LV size and function in 60 patients--20 with a normal RV, 20 with ASD or partially anomalous pulmonary venous connection, and 20 with repaired TOF [123]. In patients with repaired TOF intraclass correlations were 0.966 for RV end-diastolic volume, 0.932 for RV end-systolic volume, 0.817 for RV ejection fraction, and 0.831 for RV mass. Theses results are in agreement with those of Grothues et al. and Hudsmith et al. [124,129]. Bland-Altman analyses showed no systematic error in measurements related to the absolute value of the measures. The reproducibility of the measurements in patients with repaired TOF was not significantly different from that in patients with normal RV or with atrial septal defect. Moreover, the reproducibility of most RV measurements did not differ significantly from that of LV measurements. In practical terms, a difference of more than 34 ml/m2 (or 24%) was less than 5% likely to be explained by interobserver variability.

### CMR in Clinical Decision Making

CMR supports clinical decision making in patients with repaired TOF by providing comprehensive anatomic and functional information on postoperative cardiovascular abnormalities. Especially important in these patients is quantitative information on RV size, global and regional RV function, LV size and function, myocardial scar, RVOT aneurysm or obstruction, valve regurgitation (especially helpful when quantified), residual intracardiac shunts, and anatomic abnormalities of the pulmonary arteries and aorta. CMR is increasingly being incorporated into clinical surveillance protocols once patients with repaired TOF reach adolescence [109]. The combination of deteriorating echocardiographic windows, nearly universal ability to tolerate the CMR examination without sedation, and the comprehensive nature of the CMR data has contributed to its broad acceptance in this patient group. In addition to identifying anatomic and functional abnormalities (Table 1), CMR's ability to quantify chamber size and function and measure blood flow is especially helpful in clinical decision-making. For example, CMR can identify and characterize branch pulmonary artery stenosis (location, diameters, and length of the narrow segment) followed by flow measurements in the MPA and branch pulmonary arteries to quantify differential pulmonary flow. A relatively mild discrepancy in pulmonary blood flow (e.g., 40% flow to one lung) in the absence of other indications for catheter or surgical intervention will likely lead the clinician to recommend expectant follow-up whereas severe discrepancy (e.g., 15% flow to one lung) will likely lead to intervention. Another example is identification of an atrial septal defect followed by measurement of the pulmonary-to-systemic flow ratio. A small shunt (e.g., pulmonary-to-systemic flow ratio of 1.3) in a patient with mild PR (e.g., 15% regurgitation fraction) and mild RV dilatation (e.g., 120 ml/m^2^) will likely lead the clinician to recommend expectant follow-up whereas the same shunt in a patient with moderate PR (e.g., 35% regurgitation fraction) and RV dilatation (e.g., 145 ml/m^2^) will prompt a discussion about transcatheter device closure of the atrial septal defect versus PVR and atrial septal defect closure.

Perhaps the most critical role of CMR in supporting clinical decisions in patients with repaired TOF is determining when to replace or insert a pulmonary valve [114]. As indicated before, the decision to insert a pulmonary valve relies on clinical assessment of symptoms and signs attributable to the cardiovascular system and on measurements of PR, biventricular size and function, shunt ratio, and several morphologic criteria (e.g., RVOT aneurysm, branch pulmonary artery stenosis, severe aortic dilatation). Although some of the information necessary for clinical decision support for PVR can be obtained by other diagnostic modalities, CMR is best suited to reliably provide most or all necessary information [97,109,114]. For example, Doppler echocardiography can distinguish between mild and severe PR [130], 3-dimensional echocardiography has the potential to measure RV size and function [131], and tissue Doppler and speckle tracking can provide information on RV function [132]; RV size and function can also be measured by ECG-gated computed tomography and nuclear ventriculography [63,133]; and cardiac catheterization can provide information about pressure and shunt [134]. However, CMR provides the most comprehensive information in a single noninvasive examination without exposure to ionizing radiation [109]. Furthermore, the reproducibility of CMR in measuring key parameters necessary to support clinical decisions in repaired TOF has been published whereas similar data from other modalities in this group of patients is scant. Finally, most investigations on prognostic implications of the various parameters used to decide when to replace or insert a pulmonary valve are based on CMR data [14,15,93-96,98,135,136].

Although CMR has become the reference standard modality for follow-up of patients with repaired TOF, specific patient groups require a multimodality approach [137]. Table 4 summarizes several scenarios, in which a combination of diagnostic modalities is required to support clinical decisions after TOF repair.

**Table 4:** Examples of scenarios requiring a multimodality diagnostic approach

	Doppler Echocardiography	Cardiac CT	Nuclear Scintigraphy	Cardiac Catheterization
All patients	• Predicted RV pressure by TR jet velocity • Valve function and interrogation of atrial and ventricular septa by color Doppler			

RV hypertension with RVOT obstruction or branch PA stenosis	Predicted RV pressure by TR jet velocity			Consider if (a) possible benefit from PA balloon dilation and/or stent; or (b) transcatheter PV implantation

RV hypertension without RVOT obstruction or branch PA stenosis				Assessment of peripheral branch PA stenoses and pulmonary vascular resistance

Branch PA stenosis without reliable pulmonary flow distribution by CMR			Lung perfusion scan	

Branch PA stenosis with ≤35% flow to one lung				Consideration of balloon dilation with or without stent placement

Contraindications to CMR or large metallic artifacts		• Quantitative evaluation of RV size and function • Anatomy of RVOT and branch PAs		

Age >40 years				Coronary angiography before PVR

Secundum ASD with systemic O_2 _saturation ≤92%				Hemodynamic assessment ± device closure

ASD = atrial septal defect; PA = pulmonary artery; PV = pulmonary valve; PVR = pulmonary valve replacement; RV = right ventricle; RVOT = right ventricular outflow tract; TR = tricuspid regurgitation

## Future Directions

Research efforts in TOF aim to address the many facets of the anomaly. Examples include elucidating the genetic and developmental etiologies of the anomaly, refining the initial surgical management to minimize late complications, defining markers of late adverse outcomes, refining the criteria for PVR to optimize late outcomes, and developing new tools to address the failing RV before and after PVR. CMR plays an important role in several of these research fronts. For example, CMR is used to evaluate the function of tissue-engineered semilunar valve implanted in the pulmonary position in sheep [138]. This and similar technologies have the potential to change the way TOF is repaired by allowing the use of myocardial patches and bioengineered valves derived from the patient's own cells to reconstruct the RVOT. CMR contributes to this line of research by allowing in-vivo evaluation of structure and function of valves and ventricles.

**Figure 21:**
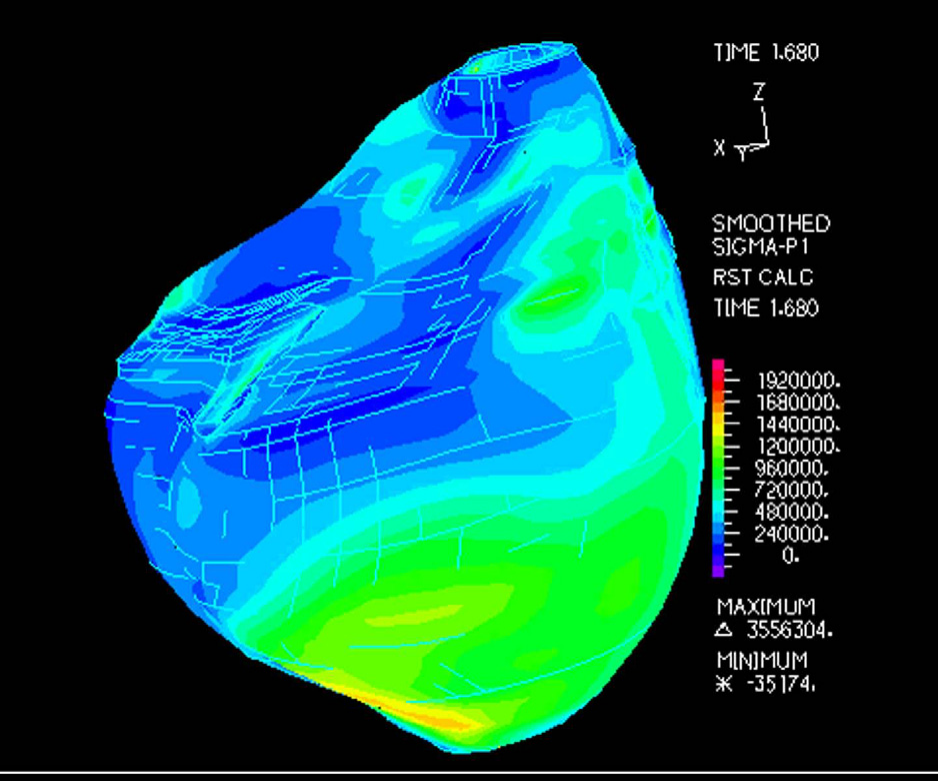
**Computer model of right ventricular stress map based on CMR data of a patient with repaired TOF**. This and similar models include fluid-structure interactions, two-layer RV-LV structure, anisotropic material properties, fiber orientation, and active contraction [139,140].

**Figure 22:**
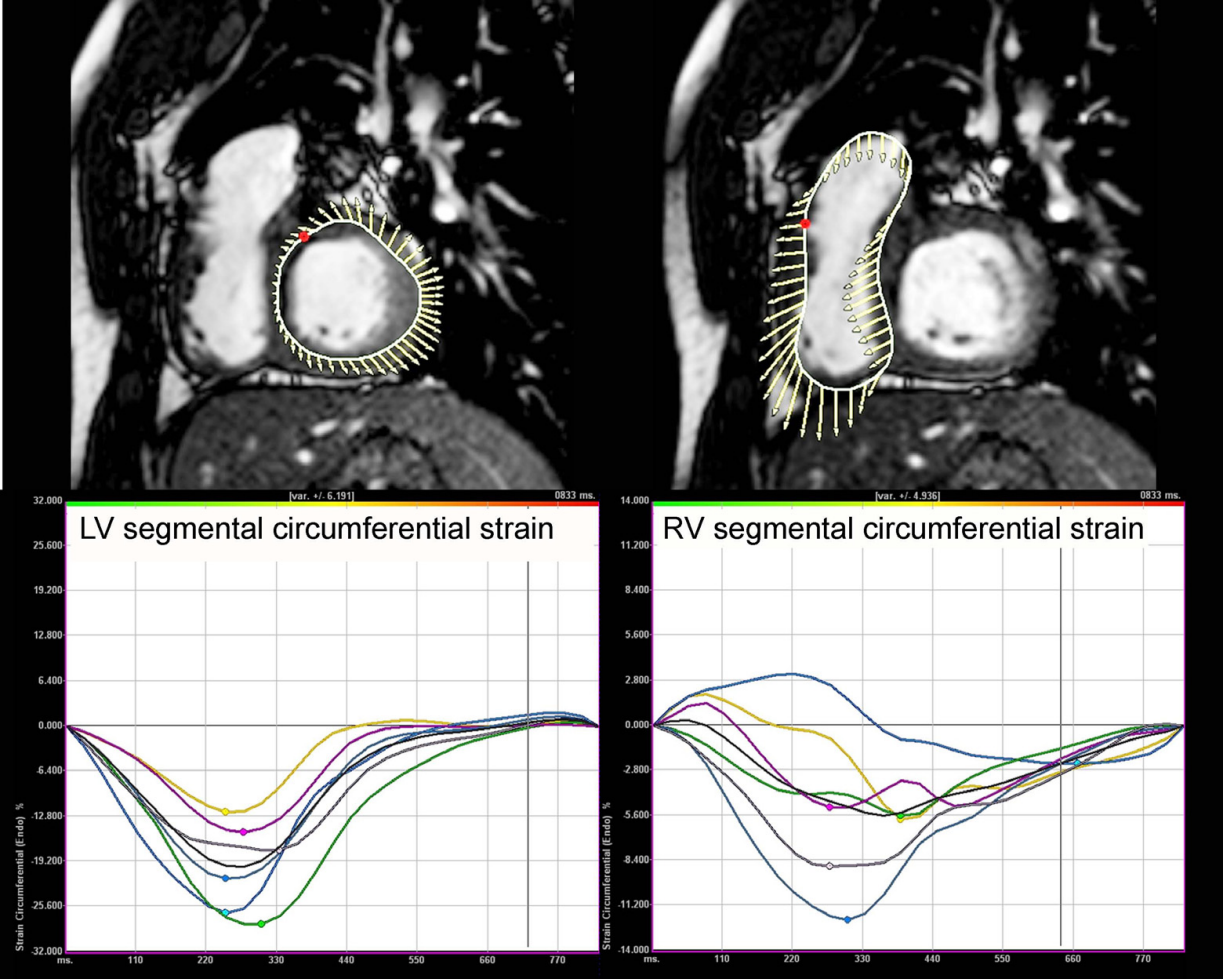
**Tissue tracking of the ventricular myocardium (LV = left panel; RV = right panel) in a patient with repaired TOF**. Analysis of circumferential strain is performed in the ventricular short-axis plane using a commercial tissue tracking software package and custom-built filters to modify the DICOM headers of the CMR datasets to allow them to be analyzed by the software package. The myocardium is divided into 6 segments and circumferential strain (Y-axis;%) versus time (X-axis; milliseconds) is plotted for each segment. The time difference to peak circumferential strain--a measure of ventricular synchrony--measured 83 ms in the LV and 389 ms in the RV, reflecting RV dyssynchrony.

Another research front in TOF is management of the failing RV late after repair. It has recently been shown that "standard" RV remodeling does not improve RV function in this group of patients [95]. Our group has conducted a series of experiments designed to develop a computational modeling approach to determine the efficacy and suitability of the various reconstructive options for the RV [139,140]. Using patient-specific CMR data of biventricular structure, deformation, and flow, RV-LV computer models were constructed. These models included fluid-structure interactions, two-layer RV-LV structure, anisotropic material properties, fiber orientation, and active contraction. The models were designed to simulate blood flow, ventricular motion, and stress-strain distribution to evaluate the effect of different remodeling procedures on RV function, and to seek an optimal RV volume and patch design to improve post-operative outcomes (Figure 21, Additional file 5). This and similar research efforts may ultimately improve surgical planning by identifying optimal individual solutions to RV remodeling based on patient-specific, CMR-derived computational models. Data from catheterization-CMR studies may provide more detailed information on RV mechanics (e.g., RV elastance or E_max_, ventricular-arterial coupling). Other advanced image analysis techniques such as CMR speckle tracking may overcome some of the limitations of RV tissue tagging and provide further insight into RV-LV mechanics in patients with repaired TOF (Figure 22) [141].

The most active clinical research efforts in repaired TOF continue to focus on refining the indications for PVR and developing better transcatheter and surgical techniques to address PR and other late complications. CMR plays a pivotal role in both fronts as the reference standard imaging tool for morphologic and functional assessments. As Table 3 demonstrates, past research efforts were dominated by small retrospective studies. Given that hard outcomes (e.g., death, ventricular tachycardia) after PVR are uncommon and it is unclear which surrogate outcomes properly reflect important outcomes, it is highly unlikely that single-center studies will provide meaningful answers. Undoubtedly, the next phase of clinical research aimed at identifying the optimal timing and method of restoring pulmonary valve competence will require analysis of large databases that include thousands patient years. This will require multicenter collaboration and, preferably, standardized prospectively acquired clinical and laboratory data, including CMR. Although a randomized clinical trial may appear to be an attractive research method to define the optimal timing of PVR, the design of such trial is challenging due to hurdles related to choice of outcomes, statistical power, and recruitment. However, once the state of knowledge in this area improves (e.g., identification of predictors of adverse outcomes after PVR and meaningful surrogate outcomes), it is conceivable that specific hypotheses can be formulated and then tested by a multicenter randomized trial. Undoubtedly, CMR will be an essential component of any such study.

## Conclusions

The pathophysiology of repaired TOF is complex. Patients often tolerate the chronic volume load imposed by pulmonary regurgitation and, in some patients, by tricuspid regurgitation and residual intracardiac shunt(s), for many years. In other patients, the pathophysiology is further complicated by abnormalities in the pulmonary arterial tree. Over time, however, the risks of ventricular dysfunction, exercise intolerance, heart failure symptoms, arrhythmias, and death increase substantially. CMR has evolved to become a crucial diagnostic tool in this growing patient population. Key to optimal use of CMR in these patients is a comprehensive imaging protocol and implementation of steps to ensure consistency and reproducibility of measurements. Finally, CMR continues to play a key role in research efforts directed at improving outcomes of patients with tetralogy of Fallot.

## Abbreviations

CMR: cardiovascular magnetic resonance; LGE: late gadolinium enhancement; LV: left ventricle; PR: pulmonary regurgitation; PVR: pulmonary valve replacement; RV: right ventricle; RVOT: right ventricular outflow tract; TOF: tetralogy of Fallot;

## Competing interests

The author declares that they have no competing interests.

## Supplementary Material

Additional file 1 **Video clip of severe RV dilatation and dysfunction after TOF repair**. Cine SSFP imaging in a 37 year-old patient with repaired TOF, severe pulmonary regurgitation, moderate tricuspid regurgitation (arrow), and severe right ventricular dilatation (end-diastolic volume index 386 ml/m^2^) and dysfunction (ejection fraction 15%). This patient who also exhibited severe heart failure symptoms had only modest decrease in RV size and no improvement in RV function after pulmonary valve replacement. Click here for file

Additional file 2 **Video clip of cine imaging of the RV long-axis**. Evaluation of the right ventricular outflow tract (RVOT) long-axis by ECG-gated cine SSFP MR. Along with the RV 2-chamber plane (Figure 10), this view demonstrates patency of the RVOT and main pulmonary artery, presence or absence of pulmonary valve tissue, and wall motion abnormalities. Click here for file

Additional file 3 **Video clip of pulmonary regurgitation after TOF repair**. Evaluation of pulmonary regurgitation (PR) by ECG-gated cine phase contrast MR. Antegrade flow from the RV to the MPA is encoded red and retrograde flow from the MPA to the RV (PR) is encoded blue. Click here for file

Additional file 4 **CMR report in repaired TOF**. Example of CMR report in a hypothetical patient with repaired TOF Click here for file

Additional file 5 **Video clip of computer modeling of the RV after TOF repair**. Computer model of right ventricular stress map based on CMR data of a patient with repaired TOF. Click here for file
