# Supplementary material for: Repaired tetralogy of Fallot: the roles of cardiovascular magnetic resonance in evaluating pathophysiology and for pulmonary valve replacement decision support
Source: J Cardiovasc Magn Reson. 2011 Jan 20;13(1):9. doi: 10.1186/1532-429X-13-9 (PMC3036629; doi:10.1186/1532-429X-13-9)
Supplement: Additional file 4 — CMR report in repaired TOF. Example of CMR report in a hypothetical patient with repaired TOF [file 1532-429X-13-9-S4.PDF]

CHILDREN'S HOSPITAL BOSTON BOSTON, MA (617) 355 8686

CARDIAC NONINVASIVE LABORATORY Record #: XX235644
CARDIAC MAGNETIC RESONANCE EXAM Name: Doe, John

Date : August 19, 2010, 00:00
Location: Outpatient Department
Doctor: Geva, Tal DVD/MOD #:
Born : July 8, 1988 Age: 22.1 years
Height: 175.0 cm Weight: 70.0 kg BSA: 1.85 m^2
Diagnosis: S/P TOF repair
Reason for test: TOF protocol

HISTORY

Tetralogy of Fallot with pulmonary stenosis, S/P repair with patch closure of the ventricular septal defect and a transannular right ventricular outflow tract patch (08/07/1990).

Cardiovascular MR examination for assessment of right ventricular size and function, pulmonary regurgitation, and branch pulmonary artery anatomy.

FINDINGS

Abdominal Situs: Solitus Cardiac Position: Levocardia
Cardiac Segments: {S,D,S} Conus: Subpulmonary

Comparison is made with the prior study on 7/8/07.

Unobstructed right ventricular outflow tract.

Dilated, unobstructed main and branch pulmonary arteries.
Differential pulmonary artery blood flow: 54% to the left pulmonary artery.

Severe pulmonary regurgitation with a regurgitation fraction of 44% by main pulmonary artery flow measurements (antegrade flow 105 ml/beat; retrograde flow 46.4 ml/beat). Prior study 41%.

Mild tricuspid valve regurgitation with 13% regurgitation fraction based on AV valve flow differential.

Dilated right ventricle (indexed end-diastolic volume 188 ml/m2; z-score= 7.25) with mild global systolic dysfunction (ejection fraction 42%). Prior study: indexed end-diastolic volume 162; z-score= 5.4; ejection fraction 47%. The anterior infundibular wall (length ~4 cm) at the presumed location of the patch is thin and dyskinetic. On myocardial delayed enhancement imaging, there is hyperenhancement in that region and a portion of the ventricular septal defect patch.

No significant right ventricular hypertension based on systolic septal configuration.

Normal left ventricular size and systolic function.

No aortic regurgitation.

Dilated aortic root and ascending aorta.

Unobstructed left aortic arch with normal branching. No significant aortopulmonary collaterals.

No residual atrial or ventricular septal defect detected; unable to exclude small defect. Qp/Qs= 1.02.

Normal systemic and pulmonary venous connections.

No pericardial or pleural effusion.

MEASUREMENTS

Heart rate (bpm):	70
Upper extremity blood pressure:	120/80, Mean = 85

Ventricular Data	Value	Z-score	(Min - Mean - Max)
LV diastolic volume (ml)	150.0	-0.07	(99 - 152 - 205)
LV systolic volume (ml)	60.0	0.36	(36 - 56 - 76)
LV ejection fraction (%)	60.0	-0.91	(55 - 64 - 73)
LV stroke volume (ml)	90.0		
LV mass (gm)	130.0	0.61	(86 - 119 - 153)
LV mass/volume (gm/ml)	0.87	0.67	(0.60 - 0.80 - 1.00)
LV ES fiberstress (gm/cm^2)	110.6		
RV diastolic volume (ml)	348.0	7.25	(108 - 159 - 210)
RV systolic volume (ml)	201.0		
RV ejection fraction (%)	42.2	-3.48	(48 - 55 - 62)
RV stroke volume (ml)	147.0		
RV mass (gm)	100.0		
RV mass/volume (gm/ml)	0.29		

Normative data for ventricular parameters by MRI from J Mag Res Imag 2003;17:323-329

Anatomic Data	A-P	R-L/S-I	Area
Aortic valve annulus (cm):	2.00	2.00	3.14
Aortic root (short-axis view, cm):	3.50	3.70	10.17
Ascending aorta (cm):	3.00	3.00	7.07
Main pulmonary artery (cm):	4.00	3.00	9.42
Right pulmonary artery (cm):	1.80	2.50	3.53
Left pulmonary artery (cm):	2.20	2.30	3.97

Flow Data	Flow	Flow/BSA

Ascending Aortic (l/min):	6.00	3.25
Main Pulmonary Artery (l/min):	6.10	3.30
Right Pulmonary Artery (l/min):	2.80	1.52
Left Pulmonary Artery (l/min):	3.30	1.79
Tricuspid Valve Inflow (l/min):	6.90	3.74
Mitral Valve Inflow (l/min):	6.00	3.25

Flow Fractions and Regurgitant Fractions

Pulmonic Regurgitant Fraction	0.44
Tricuspid Regurgitant Fraction	0.13

CARDIOLOGY CODES AND INTERPRETATION

NORMAL PULMONARY VENOUS CONNECTIONS	300800
TRICUSPID REGURGITATION, MILD	173001
RIGHT VENTRICULAR DYSFUNCTION, MILD	182201
RIGHT VENTRICULAR DYSFUNCTION REGIONAL	182400
LEFT VENTRICULAR DYSFUNCTION RULED OUT	181208
TETRALOGY OF FALLOT	105000
PULMONARY REGURGITATION, SEVERE	165003
DILATATION OF THE MAIN PULMONARY ARTERY	232400
LEFT AORTIC ARCH	271000
NORMAL ORIGIN OF THE RIGHT AND LEFT CORONARY ARTERIES	309800
S/P VSD CLOSURE, VALVOTOMY AND TRANSANULAR OUTFLOW PATCH FOR TOF	544600

TECHNIQUE

Scanner: Cardiac Gating: ECG Coil: 32C-Cardiac
Sedation: None
Gadolinium dose (cc): 28.0
Sequences:

3-plane and interactive localizing SSFP sequences. ECG-gated, breath-hold SSFP cine: ventricular long- and short-axis planes; right ventricular outflow tract long-axis; and axial plane across the pulmonary arteries. Gadolinium-enhanced 3D magnetic resonance angiogram. ECG-gated, breathe-through phase velocity flow measurements: aortic root, main pulmonary artery, branch pulmonary arteries, and atrioventricular valves. Myocardial delayed enhancement imaging in ventricular long- and short-axis planes.

E-signed August 20, 2010, 01:35 PM Tal Geva, M.D.
